# Synthetic Cells Revisited: Artificial Cells Construction Using Polymeric Building Blocks

**DOI:** 10.1002/advs.202305837

**Published:** 2023-11-20

**Authors:** Viviana Maffeis, Lukas Heuberger, Anamarija Nikoletić, Cora‐Ann Schoenenberger, Cornelia G. Palivan

**Affiliations:** ^1^ Department of Chemistry University of Basel Mattenstrasse 22 Basel CH‐4002 Switzerland; ^2^ NCCR‐Molecular Systems Engineering BPR 1095, Mattenstrasse 24a Basel CH‐4058 Switzerland; ^3^ Swiss Nanoscience Institute University of Basel Klingelbergstrasse 82 Basel CH‐4056 Switzerland

**Keywords:** artificial cells, artificial organelles, artificial signaling, bottom–up, collective behavior, communicative networks, polymers

## Abstract

The exponential growth of research on artificial cells and organelles underscores their potential as tools to advance the understanding of fundamental biological processes. The bottom–up construction from a variety of building blocks at the micro‐ and nanoscale, in combination with biomolecules is key to developing artificial cells. In this review, artificial cells are focused upon based on compartments where polymers are the main constituent of the assembly. Polymers are of particular interest due to their incredible chemical variety and the advantage of tuning the properties and functionality of their assemblies. First, the architectures of micro‐ and nanoscale polymer assemblies are introduced and then their usage as building blocks is elaborated upon. Different membrane‐bound and membrane‐less compartments and supramolecular structures and how they combine into advanced synthetic cells are presented. Then, the functional aspects are explored, addressing how artificial organelles in giant compartments mimic cellular processes. Finally, how artificial cells communicate with their surrounding and each other such as to adapt to an ever‐changing environment and achieve collective behavior as a steppingstone toward artificial tissues, is taken a look at. Engineering artificial cells with highly controllable and programmable features open new avenues for the development of sophisticated multifunctional systems.

## Graphical abstract

The surge in artificial cell and organelle research underscores their potential to advance their understanding of fundamental biology. This review centers on crafting artificial cells from micro‐ and nanoscale polymer‐based compartments, harnessing their varied chemistry for customizable assembly. The authors delve into supramolecular architectures, membrane types, supramolecular structures, functional attributes, communication, and collective behavior, illuminating pathways for building artificial tissues.

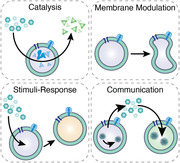

## 1 Introduction

The complexity and multifunctionality of cells and the changes that arise under pathologic conditions inspired researchers to create artificial organelles and cells. They are intended as models to understand bio‐processes, signaling and communication,^[^ ^1^, ^2^, ^3^, ^4^, ^5^ ^]^ to provide diagnostics and treatment solutions,^[^ ^6^, ^7^ ^]^ and to develop new materials with close‐to‐nature functionality.^[^ ^7^, ^8^, ^9^, ^10^ ^]^ By means of a bottom–up strategy cellular processes are broken down into simpler pathways and reactions to eventually recapitulate them in user‐defined compartments under controlled conditions, removed from the intricate environment of natural cells.^[^ ^11^, ^12^, ^13^, ^14^, ^15^ ^]^ Similarly, artificial organelles as sub‐compartments of cells are designed in a bottom–up approach by encapsulating/entrapping active compounds (enzymes, proteins, DNA, mimics of thereof) inside nanoassemblies (micelles, vesicles, nanoparticles, hydrogels) that will be sequestered and protected from the surrounding while performing their intrinsic activity/functionality.^[^ ^12^, ^16^, ^17^, ^18^ ^]^ Another key aspect to be taken into account when designing reactive artificial organelles and cells is the transmission of molecules and signals between compartments.

Lipid‐based compartments (liposomes and giant unilamellar vesicles, respectively) have been largely used for the development of artificial cells and organelles because the phospholipid composition of their membrane compares to that of natural cells.^[^ ^19^, ^20^, ^21^ ^]^ Many excellent reviews have been published recently that address the bottom–up construction of artificial cells based on giant unilamellar lipid vesicles (l‐GUVs).^[^ ^22^, ^23^, ^24^, ^25^, ^26^, ^27^ ^]^ However, the intrinsic mechanical instability and presence of defects limits the use of l‐GUVs for advanced applications and motivated researchers to search for more robust compartments. Synthetic amphiphilic polymers are the building blocks of choice because they allow formation of nano‐ and micro‐compartments with,^[^ ^28^ ^]^ improved properties compared to the lipid‐based ones (e.g., mechanical stability, thickness, and permeability of the membranes). In addition, polymer building blocks can be tuned to confer special features such as stimuli‐responsiveness, shape plasticity, or self‐organization into clusters upon the ensuing compartments.^[^ ^23^, ^24^, ^25^, ^26^, ^27^ ^]^ While the overall hollow spherical shape is similar for lipid and polymer compartments, the difference between their membrane properties arises from the divergent molecular weight of their hydrophilic and hydrophobic domains. Importantly, this difference governs the combination with biomolecules and presents many challenges regarding the preservation of biomolecular integrity and/or functionality in synthetic compartments. To create cell‐like compartments, amphiphilic copolymers self‐assemble into micrometer‐sized compartments (synthetic or p‐GUVs) with simultaneous encapsulation of nanoassemblies (lipid‐ or polymer‐based) and active biomolecules.^[^ ^14^, ^15^, ^33^ ^]^ The result is a “compartments‐in‐compartment” architecture that mimics in a simplified manner the presence of organelles inside a cell. This multi‐compartmentalization allows for active compounds within different nanocompartments to participate in simple reactions as part of metabolic pathways for longer periods of time than is the case for lipid‐based artificial cells. In addition, the synthetic cells with polymer components allow to introduce stimuli‐responsive features or even new‐to‐nature reactions between the compartments.^[^ ^20^, ^34^, ^35^ ^]^ While many compartments‐in‐compartment polymer systems are membrane enclosed, a membrane‐less confinement is becoming popular as different type of artificial cell.^[^ ^36^, ^37^, ^38^, ^39^ ^]^

**Figure 1:**
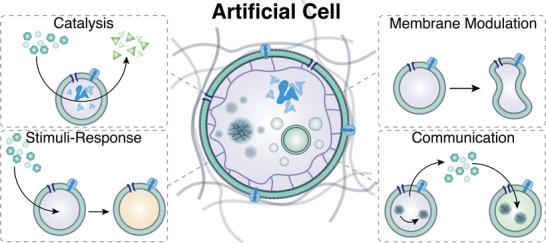
The artificial cell possesses the capacity to emulate the structure of a biological cell in a simplified manner. Its hierarchical multi‐compartmentalization enables active compounds within distinct nanocompartments to engage in straightforward cascade reactions and communication when organized within cell consortia.

In this review, we first explore nano‐ and microscale polymer assemblies and how they are used as building blocks for artificial cells (**Figure** **1**). For the purpose of this review, we use artificial cell, synthetic cells, and protocell interchangeably. We mostly focus on cell‐sized compartments where polymers are the major constituent of the assembly because owing to their improved properties and an unprecedented spatiotemporal control of a desired function, they uniquely support advanced applications over long time periods. An additional benefit is that they can be furnished with new‐to‐nature behavior. For nanosized compartments, we consider mainly bottom–up produced assemblies of nonbiological and/or biological components that are designed to recapitulate one or more features of biological cells. In particular, we present different membrane‐bound and membrane‐less compartments and supramolecular structures based on polymers, and briefly summarize how they are produced, before describing how they turn into functional modules mimicking cellular processes. Nanosized assemblies that are customized to mimic features of natural cells but also those that, once internalized, provide living cells with a distinct activity we define as artificial organelles. We address how artificial organelles convert polymer‐based giant compartments into sophisticated multifunctional synthetic cells. We further explore special cell behaviors, properties, and communication that provide first insights into collective features and support development of multifunctional materials. The large variety of polymer‐based artificial organelles and cells lend themselves to exploring the function of biomolecules in synthetic environments, and eventually offer cutting‐edge solutions in various domains, including medicine, catalysis, technology. However, due to the incredible complexity of natural cells, there are still a plethora of challenges to overcome and we will point at some of them as an outlook for new avenues for the field to explore.

## 2 Building Blocks for the Generation of Artificial Cells

### 2.1 Membrane‐Bound Micro‐Compartments as Cell Mimics

**Figure 2:**
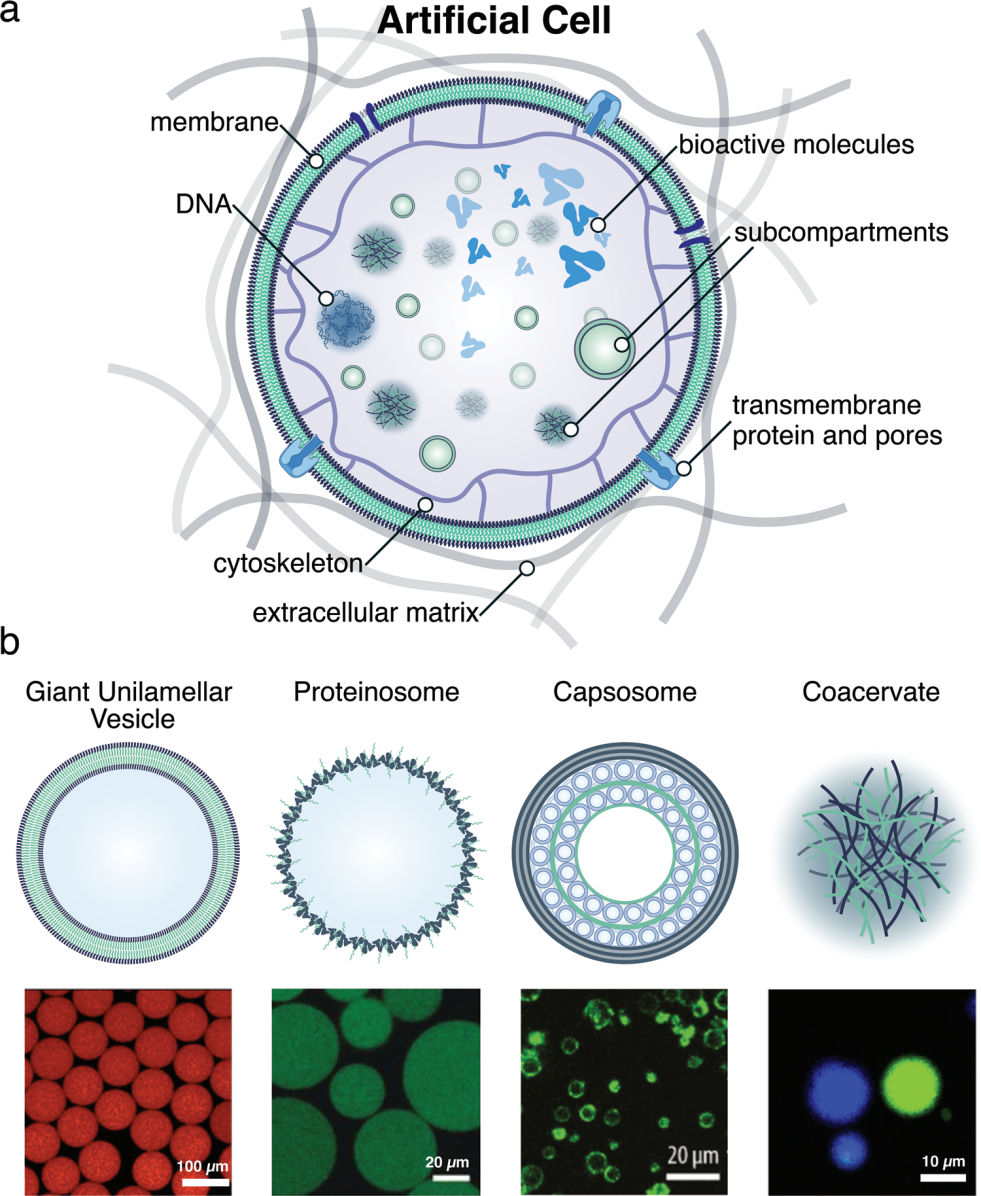
a) Schematic illustration of a large outer micro‐compartment with incorporation of hierarchical systems and their organization in functional membrane bound or membrane less compartments. b) Schematic overview of polymeric based architecture used for cell mimics. Reproduced with permission.^[^ ^72^, ^77^, ^79^, ^80^ ^]^ Copyright 2019, Wiley‐VCH. Copyright 2016, American Chemical Society. Copyright 2019, American Chemical Society. Reproduced with permission under terms of the CC‐BY‐NC‐ND license.^[^ ^72^ ^]^ Copyright 2017, American Chemical Society.

An essential starting point for building a simple cell model is to generate a micrometer‐sized compartment surrounded by a bilayer membrane (**Figure** **2**). The demands for mimicking natural cell membranes are manifold: the polymer boundary should be selectively permeable, host multifunctional membrane proteins, enable bidirectional flow of ions and molecules, create temporary gradients to function under out‐of‐equilibrium conditions, mediate interactions of cells with their environment, and at the same time be sufficiently flexible to afford shape changes. The plasma membrane encloses a variety of organelles and biomolecules that, for participating in different metabolic and signaling pathways, need to be organized in a dynamic and adaptive architecture. As these membrane features are dictated by many regulatory pathways in a dissipative, far‐from‐equilibrium manner, artificial cells cannot yet mimic intricate behaviors. In an artificial cell, the boundary does not only function as barrier, but also provides a matrix for membrane proteins and acts as an anchor site for molecules involved in external and internal communication.^[^ ^40^ ^]^ Considerable research efforts are directed toward constructing cell‐sized compartments based on various components including phospholipids, block copolymers, and protein–polymer hybrids with corresponding membrane properties and functionalities. The assembly of the building blocks results in different supramolecular assemblies: i) giant vesicles enclosed by a single membrane and ii) giant vesicles enclosed by a multi‐layered membrane. The wide variety of micrometer‐sized compartments confine different reactants (proteins, enzymes, mimics of thereof) and allow them to act in situ while being protected from harsh external environments. Encapsulated active compounds are capable of replicating specific biochemical processes and have been exploited to advance various processes and applications, such as signaling,^[^ ^15^, ^41^ ^]^ cytoskeleton assembly,^[^ ^34^, ^42^ ^]^ drug delivery,^[^ ^2^ ^]^ or the printing of artificial tissues.^[^ ^43^, ^44^ ^]^ A more complex performance of microcompartments is achieved by encapsulation of distinct nanoassemblies (polymersomes, nanoparticles, micelles and combinations of thereof) which results in a compartments‐in‐compartment architecture that mimics the hierarchical architecture of eukaryotic cells.^[^ ^15^, ^33^, ^45^ ^]^

#### 2.1.1 Giant Vesicles with a Single‐Layer Boundary

Giant vesicles with a single‐layer boundary are micrometer‐sized compartments enclosed by a self‐assembled bilayer of lipids, polymers or hybrids thereof (giant unilamellar vesicles) or by association of protein–polymer hybrid amphiphiles (proteinosomes).

#### 2.1.2 Giant Unilamellar Vesicles

Spherical, micrometer‐sized compartments termed giant unilamellar vesicles (GUVs) are the prevalent type of synthetic cell. To assemble GUVs, the building blocks can vary from phospholipids, amphiphilic polymers, and hybrids thereof. Their physico‐chemical properties, for example, molecular weight, charge, amphiphilicity, and flexibility determine the resulting membrane structure. In addition, they influence membrane features such as mechanical stability, permeability and lateral fluidity, which play a critical role when it comes to the insertion of biomolecules (biopores or membrane proteins). GUVs per se lack a chemically enriched, molecularly crowded interior, which is a hallmark of cellular organization. However, synthetic cells based on GUVs can be engineered at high organizational complexity and multifunctionality by encapsulating functional nanomodules mimicking natural organelles and/or active compounds (enzymes, proteins, catalysts) during their formation process.

Polymer‐based giant unilamellar vesicles (p‐GUVs) are self‐assembled, cell‐sized compartments (1–100 µm) delineated by an amphiphilic block copolymer membrane.^[^ ^3^ ^]^ The vesicular structure is characterized by a single bilayer with a central hydrophobic domain between two hydrophilic domains serving as interfaces, one toward the inner aqueous cavity and the second one facing the external aqueous environment. Owing to the similarity of the synthetic bilayer morphology to that of natural lipid membranes, p‐GUVs were identified as biomimetic structures that lend themselves to biophysical investigations on principles governing membrane formation and interactions and to the construction of cell‐like systems that imitate basic functions of living cells.^[^ ^30^ ^]^ Notably, although amphiphilic block copolymers and phospholipids have a similar architecture in terms of a hydrophobic and at least one hydrophilic domain, the molecular weight of block copolymers are orders of magnitude larger than those of lipids. Thus, the membrane thickness of the resulting GUVs ranges from 5 to 50 nm for block copolymers whereas lipid bilayers are 3–5 nm thick on average.^[^ ^46^ ^]^ As a consequence, features such as membrane permeability, viscosity and rigidity and mechanical stability of p‐GUVs significantly differ from those of l‐GUVs which are vulnerable structures with short lifetimes.^[^ ^32^ ^]^ For instance, the thicker membrane of p‐GUVs is associated with a low permeability unless copolymers were specifically selected to assemble an intrinsically porous membrane.^[^ ^47^ ^]^ Overall, the enhanced mechanical stability is an asset of polymer membranes although it makes the insertion of biomolecules such as biopores or membrane proteins more challenging.^[^ ^40^, ^48^ ^]^ Therefore, depending of the desired properties of the resulting p‐GUVs, the amphiphilic copolymers should be appropriately selected in terms of the chemical nature, block length and hydrophilic‐to‐hydrophobic ratio.^[^ ^15^, ^34^, ^49^ ^]^ The compositional flexibility and enhanced stability of p‐GUVs make them highly promising for biotechnological or biomedical applications.

Hybrid giant unilamellar vesicles have been developed to combine advantageous features of different building blocks and thereby expand the complexity of the synthetic membrane. Research involving hybrid giant vesicles has mainly focused on compartments derived from lipid‐copolymer mixtures.^[^ ^50^, ^51^ ^]^ A key aspect in the formation of stable hybrid lipid/copolymer vesicles is to avoid a high discrepancy in the length of the hydrophobic domains between lipids and polymers by selecting components with appropriate chemical structure.^[^ ^50^ ^]^ When forming GUVs from pure lipids, the interactions between the lipid tails do not strongly affect the assembly process. On the contrary, in the case of lipid/copolymer mixtures, the nature of the monomeric unit of the copolymer hydrophobic block may lead to immiscibility between the copolymer block chains and the phospholipid tails, resulting in unstable vesicles.^[^ ^52^ ^]^ Overall, strong geometric differences between the lipids and polymer and thermodynamic incompatibility arising from both entropic and enthalpic differences between the hydrophobic blocks may induce phase separation and ultimately compromise hybrid polymer/lipid vesicle formation.^[^ ^46^, ^50^ ^]^

#### 2.1.3 Proteinosomes

Proteinosomes are higher‐order structures with a compartmentalized architecture based on the spontaneous assembly of amphiphilic protein‐polymer nanoconjugates at the water/oil (W/O) interface of a pickering emulsion.^[^ ^53^, ^54^ ^]^ In contrast to the relatively inert membranes of p‐GUVs, the proteinaceous boundary of proteinosomes can be prepared from different enzyme‐NH_2_‐polymer building blocks capable of carrying out multi‐step membrane‐mediated cascade reactions.^[^ ^55^ ^]^ Typically, proteinosome membranes show good permeability which enables the exchange of biomolecules with a molecular weight up to ≈10 kDa. Based on their unique properties, proteinosomes serve as biomimetic microcompartments that, depending on the set of external and enclosed biomolecules, support different cellular processes including gene‐directed protein synthesis or internal enzyme‐mediated transformations.^[^ ^55^, ^56^ ^]^ Recent advancements have demonstrated that by selecting corresponding nanoconjugates and cross‐linker, the permeability of proteinosome membranes could be sequentially modulated by changes in temperature, redox species, and pH.^[^ ^57^ ^]^ Developing responsive proteinosomes with greater precision and adaptability to environmental conditions holds significant promise for constructing protocells with advanced features including regulated chemical communication and control over metabolic reactions.^[^ ^58^ ^]^

Actin, a naturally occurring polymer, when appropriately conjugated, can create artificial compartments in the range of 2–5 µm known as Actinosomes.^[^ ^59^ ^]^ These structures possess several properties that make them potentially valuable for synthetic cell research. Notably, they are formed from proteinaceous building blocks which makes them biocompatible and biodegradable.

#### 2.1.4 Giant Capsules with a Multi‐Layer Boundary

Capsosomes are hierarchical structures comprised of liposomes incorporated in a polymer capsule which are particularly interesting for the creation of artificial cells.^[^ ^60^ ^]^ Formed through alternating layers of oppositely charged liposomes and polymers, these assemblies possess remarkable application potential by merging the advantages of two distinct systems within these compartmentalized assemblies.^[^ ^61^ ^]^ While the polymer capsule imparts structural integrity to the construct and governs the diffusion of (bio)molecules through the permeable polymer membrane, liposomes mimic cellular organelles, offering specialized subunits that when loaded with enzymes facilitate corresponding enzymatic reactions within a confined region. Moreover, by engineering capsosomes, the capability to regulate the number of subcompartments can be achieved, thus controlling cargo loading levels.^[^ ^62^ ^]^ So far, nondegradable,^[^ ^63^ ^]^ and (bio)degradable,^[^ ^64^ ^]^ carrier capsules containing liposomes have been reported. Furthermore, the effective retention of enzymatic cargo within these liposomal subcompartments,^[^ ^65^ ^]^ along with the successful execution of a controlled enzymatic reaction upon the dissolution of the liposomes, has been achieved.^[^ ^66^ ^]^ In addition, capsosomes were shown to be suitable for encapsulating small molecules and therapeutics.^[^ ^64^, ^67^, ^68^ ^]^ The multi‐layered structure of capsosomes offers adaptable drug‐loading potential, making them highly suitable for delivering multiple drugs simultaneously. The liposome components favorably encapsulate hydrophilic molecules in the aqueous lumen while hydrophobic compounds are accommodated within the liposomal membrane.^[^ ^64^, ^69^ ^]^

### 2.2 Membrane‐Less Microcompartments: Coacervates

The significance of membrane‐less compartments (or coacervates) for protocell construction lies in their resemblance to biomolecular condensates with specific functions observed in living organisms.^[^ ^70^ ^]^ One of the key characteristics of these membrane‐less compartments is their formation through liquid‐liquid phase separation, leading to the creation of a condensed phase and a dilute phase.^[^ ^37^ ^]^

Coacervates create a dense, charged, and crowded microenvironment that facilitates the reconstruction and study of basic biological processes by combining polycations and polyanions, such as poly‐l‐lysine and RNA.^[^ ^71^ ^]^ However, the membrane‐free nature of complex coacervates limits their applicability to conditions that require extended time scales.^[^ ^72^ ^]^ The electrostatic interactions that drive the condensation process also promote rapid coalescence and loss of structural morphology. To overcome this limitation, researchers have found ways to stabilize complex coacervates using various agents, including phospholipids,^[^ ^73^ ^]^ phosphotungstate polyanionic clusters,^[^ ^74^ ^]^ and triblock copolymers.^[^ ^72^ ^]^ These stabilizing agents create a semi‐permeable compartment boundary, allowing for the encapsulation of functional macromolecules while preventing coalescence and content mixing. For example, coacervates separated from the environment by a self‐assembling polymer membrane, was demonstrated for poly(ethylene glycol), poly(caprolactone‐gradient‐trimethylene carbonate), and poly(glutamic acid) (PEG‐PCL*g*TMC‐PGlu) on amylose‐based coacervates.^[^ ^72^, ^75^, ^76^ ^]^ Other methods to separate them from the environment include encapsulation of coacervates in spontaneously assembled proteinosomes,^[^ ^77^, ^78^ ^]^ or in p‐GUVs.^[^ ^42^ ^]^

### 2.3 Nanoassemblies as Mimics of Natural Organelles

Artificial organelles are nanoassemblies engineered to either mimic functions of natural organelles or to provide non‐native activity when taken up by biological cells.^[^ ^81^, ^82^, ^83^ ^]^ The incorporation of artificial organelles into cells is particularly appealing as novel medical paradigm in the treatment of various cell disorders associated with malfunctioning organelles. The chemical nature of the components forming nanoassemblies is very diverse, as are their architectures. In the following, we describe: i) polymersomes, ii) micelles, iii) nanoparticles, and iv) hydrogels as the most prominent examples mimicking cellular subcompartments. We will briefly discuss clusters of nanoassemblies in the context of mimicking organelle interactions.

#### 2.3.1 Polymersomes

Polymersomes, known for their vesicular nature and structural stability, are currently the most popular nanoassemblies used for mimicking natural organelles.^[^ ^30^, ^84^ ^]^ As hollow spherical compartments delimited by a bilayer of block copolymers, they have the advantage of a dual carrier role—they can serve as host to hydrophilic molecules inside their cavities and/or to hydrophobic molecules in their membranes.^[^ ^48^, ^85^ ^]^ Polymersomes, due to their structural stability, are particularly suited to protect encapsulated enzymes such that they preserve their in situ reactivity over long periods of time.^[^ ^86^ ^]^ Polymersome membranes usually exhibit low permeability unless they are assembled from selected polymers whose chemical nature results in an inherently porous membrane.^[^ ^87^ ^]^ For selective permeability, the incorporation of natural channels into the membrane or of chemically/genetically‐modified ones to respond to the presence of external stimuli is required to facilitate access of substrates to the encapsulated enzymes.^[^ ^88^, ^89^ ^]^ Different methods for permeabilizing polymer membranes are discussed below.

#### 2.3.2 Micelles

At different hydrophobic to hydrophilic block ratios, amphiphilic block copolymers self‐assemble into micelles that have a hydrophobic core surrounded by a hydrophilic corona.^[^ ^90^, ^91^ ^]^ Micelles are easily assembled into monodisperse populations with inherently small sizes, typically below 100 nm, making them excellent candidates for therapeutics. Although polymeric micelles have demonstrated their potential as drug carriers in clinical applications, they are relatively underexplored as artificial organelles.^[^ ^92^ ^]^ One example proposed as artificial organelle involved micelles containing salen–manganese complexes which exhibit catalase‐mimetic activities and acted as scavenger for reactive oxygen species (ROS) in HepG2 cells.^[^ ^93^ ^]^ This example paves the way for further exploration of polymeric micelles as functional components in artificial cells.

#### 2.3.3 Polymeric Nanoparticles

Responsive polymeric nanoparticles, which have a hard core, are mainly proposed for the delivery of bioactive compounds, including drugs, proteins, genes, and nucleic acids. Their notable trait lies in their capacity to break down and liberate their payload in response to various triggers, including endogenous stimuli (pH, enzymes, temperature, redox potential, hypoxia, glucose levels) or exogenous stimuli (light, magnetism, ultrasound, electrical pulses) for the controlled release of drugs or genes at designated locations.^[^ ^94^, ^95^ ^]^ In the field of artificial organelles they have been only recently used as subcompartments in p‐GUVs.^[^ ^15^ ^]^ By taking advantage of the selective permeability of the p‐GUV membrane for dithiothreitol (DTT), a reducing environment was generated in the p‐GUVs' cavity by diffusion of externally added DTT. The reductive milieu caused the reduction‐sensitive nanoparticle subcompartments to disintegrate, leading to the release of the biomolecules entrapped inside and the start of the desired sequence of reactions.

#### 2.3.4 Nano‐Sized Hydrogels

Using hydrogels to compartmentalize artificial cells is another relatively recent approach.^[^ ^36^, ^96^ ^]^ Nano‐sized hydrogels, so‐called nanogels, are 3D networks of crosslinked polymers that can absorb and store large amounts of water due to the physical and chemical cross‐linking of the polymer chains. Being composed of nearly 95% water, hydrogels offer a similar environment to natural cells, making them suitable for artificial cell applications.^[^ ^97^ ^]^ The sheer limitless complexity and versatility of hydrogel constituents allows for customization of their properties and novel applications. DNA in particular is being increasingly used to produce functional and responsive hydrogels, with huge potential in synthetic cell engineering.^[^ ^98^, ^99^, ^100^ ^]^ They provide structure and compartmentalization, enabling both intracellular and extracellular behavior.^[^ ^36^ ^]^ Compartmentalization within nanogels creates specific areas or phases with designed properties, allowing for cargo encapsulation,^[^ ^101^ ^]^ tailored cargo release,^[^ ^102^ ^]^ and support for chemical reactions.^[^ ^103^ ^]^ This versatility allows a wide range of functions to be implemented in nanogels, mimicking internal organelles found in biological cells.^[^ ^104^ ^]^ In more complex systems, enzymes can be incorporated into chemically distinct hydrogel compartments, enabling spatially separated interactions and system‐level tandem reactions that imitate cellular behavior.^[^ ^105^ ^]^ Recent example include the design of a nucleus‐like DNA‐hydrogel compartment, capable of expressing proteins and communicating with neighboring cell‐mimics through diffusive protein signals.^[^ ^106^ ^]^ These advancements hold great promise for the development of sophisticated artificial cells with diverse applications in biotechnology and medicine.

#### 2.3.5 Clustered Nano‐Assemblies

Similar to closely apposed natural organelles that communicate, the design of clusters of subcompartments is meant to allow more rapid communication and efficient reactions between separate nano‐spaces. In particular, DNA, due to the precise sequence programmability, unique molecular recognition ability, and good biocompatibility, has been explored as superior building blocks to tether together artificial organelles. By exploiting the propensity of complementary DNA strands to hybridize, polymersomes modified with complementary DNA strands have been spatially integrated to mimic the connections of natural organelles.^[^ ^29^, ^81^ ^]^ Clustered polymersomes served as platforms for cascade reactions, where different enzymes were encapsulated in separate polymersomes within the clusters, enabling controlled and spatially organized enzymatic reactions.^[^ ^81^ ^]^ Furthermore, the interaction of polymersome clusters with various cell lines has shown the effect of specific cell membrane receptors on the fate of the clusters that were either docked on the cell membrane or taken up by cells.^[^ ^107^ ^]^ Of particular interest was to use strain‐promoted azide‐alkyne cycloaddition (SPAAC) click cross‐linking to attach linkers, such as ssDNA, on preformed polymersomes which then favor the clustering process, and for fabrication of artificial organelles facilitating spatially controlled enzymatic reactions.^[^ ^81^, ^108^ ^]^ This approach avoids DNA prefunctionalization of the block copolymers, which would alter the ratio between hydrophilic and hydrophobic blocks and subsequently interfere with the self‐assembly process.

## 3 Methods for Generating Artificial Organelles and Cells

### 3.1 Criteria for Polymer Selection

The copolymer used for building an artificial cell by self‐assembly largely influences the properties of the resulting synthetic compartment and thus its ability to mimic a natural cell. Among the most important requirements is biocompatibility. It is essential that copolymers interface efficiently with bioactive components such that their native structure and function are preserved. This also applies to the solvents chosen for the generation of nano‐ and micro‐compartments in order to not affect the biomolecule constituents which bestow the characteristics of distinct organelles and cells upon the compartments.

Another important factor in the selection of the copolymers is the presence of specific functional groups that allow the coupling of bioactive components, such as enzymes, proteins, or nucleic acids, to the polymers without the use of harsh chemical crosslinking methods. Biocompatible attachment of biological components to the synthetic membranes includes copper‐catalyzed azide‐alkyne cycloaddition (CuAAC),^[^ ^109^, ^110^ ^]^ or its copper‐free counterpart, SPAAC,^[^ ^81^, ^111^, ^112^, ^113^ ^]^ EDC‐NHS immobilization,^[^ ^112^ ^]^ maleimide crosslinking,^[^ ^114^ ^]^ or DNA‐mediated functionalization.^[^ ^81^ ^]^ As described above, these coupling methods have also been used for clustering membraned‐bound subcompartments.

In addition, the copolymers should be stable under the conditions used for their self‐assembly, in particular they should exhibit thermal stability and resistance to degradation under physiological conditions. In general, this is not a problem for polymeric compartments as they are more stable than lipid vesicles, which is related to the physicochemical properties of the polymer building blocks. These in turn determine the properties of the assembled membrane such as permeability, mechanical strength, and flexibility which have a significant impact on the behavior and functionality of the artificial cell. For example, membrane flexibility is an important parameter for inserting membrane proteins even in thick membranes as in the case of synthetic ones.^[^ ^115^ ^]^

Furthermore, a defined control of membrane permeability is critical for artificial cell function. While liposomes are mostly inherently permeable to small molecules, the permeability of polymeric compartments can be tailored for specific applications. Intrinsically permeable polymers such as Pluronic L121,^[^ ^42^, ^79^ ^]^ and oligo(aspartic acid)‐*b*‐poly(propylene oxide),^[^ ^116^ ^]^ are convenient solutions to overcome diffusion limitations or impermeability of membranes. However, this permeability can affect GUV stability and does not afford precise control over the diffusion of different molecules across the membrane.^[^ ^117^, ^118^ ^]^ Membranes with selective permeability where only desired molecules pass through are often preferable as they more closely mimic natural cell membranes.^[^ ^40^ ^]^ Selective permeability of synthetic membranes has been achieved by the insertion of ionophores,^[^ ^48^ ^]^ pore forming peptides,^[^ ^88^ ^]^ membrane proteins,^[^ ^119^, ^120^, ^121^ ^]^ and DNA Origami pores.^[^ ^122^, ^123^ ^]^

### 3.2 Production of Micro‐ and Nanocompartments Delimited by Membranes

#### 3.2.1 Polymersomes and Polymer Giant Unilamellar Vesicles

**Figure 3:**
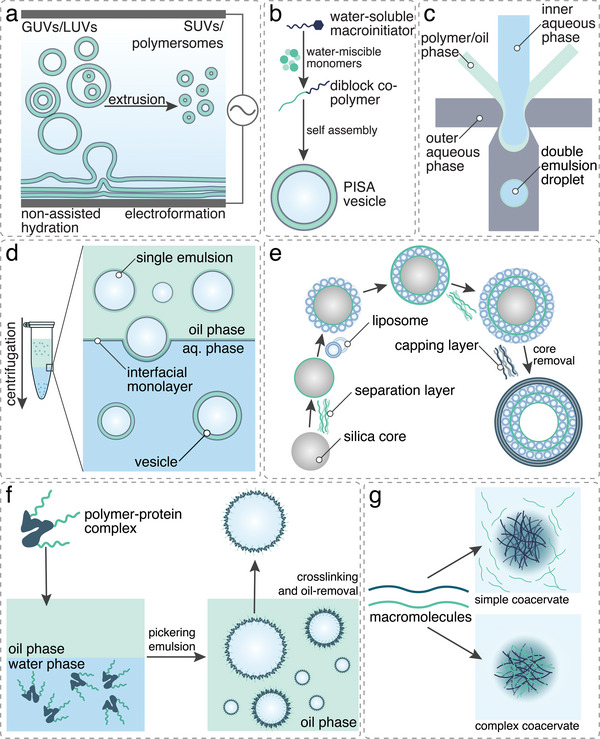
Schematic representation of selected production methods for artificial cells and organelles: a) film rehydration, electroformation, and subsequent extrusion of block copolymers, b) polymerization‐induced self‐assembly (PISA), c) double emulsions microfluidic formation of vesicles, d) emulsion centrifugation, e) layer‐by‐layer assembly of capsosomes, f) pickering emulsion self‐assembly of proteinosomes, and g) self‐assembly of coacervates.

Microscale p‐GUVs and nanoscale polymersomes and micelles are generated by self‐assembly of amphiphilic copolymers or mixtures thereof with lipids, proteins, or peptides (**Figure** **3**).

There are various methods to generate compartments with vesicular architecture, including film rehydration,^[^ ^124^, ^125^ ^]^ and microfluidic double emulsion techniques.^[^ ^79^, ^119^ ^]^ Each of these approaches offers unique advantages in terms of throughput, control over vesicle size, and encapsulation of molecules and subcompartments.

Film rehydration is based on dissolving block copolymers in a volatile, often organic solvent that is completely evaporated and forms a thin copolymer film on a surface. This film is subsequently rehydrated with an aqueous solution, often a buffer, which induces the self‐assembly of the block copolymers and formation of distinct supramolecular assemblies such as micelles, vesicles, and worm‐like assemblies.^[^ ^126^ ^]^ Film rehydration method is used to prepare polymersomes,^[^ ^127^, ^128^, ^129^ ^]^ p‐GUVs,^[^ ^124^ ^]^ and hybrid GUVs.^[^ ^130^ ^]^ The film rehydration method is suitable for a wide range of copolymers, such as poly(dimethylsiloxane)‐poly‐*b*‐(methyloxazoline),^[^ ^15^, ^34^, ^88^ ^]^ poly(ethylene oxide)‐*b*‐poly(acrylic acid),^[^ ^131^ ^]^ polybutadiene‐*b*‐poly(ethylene ethyl phosphate) or polybutadiene‐*b*‐poly(ethylene ethyl phosphate.^[^ ^125^ ^]^ It has also been further applied to hybrid polymer‐lipid mixtures,^[^ ^132^ ^]^ and amphiphilic elastin‐like polypeptides (ELPs).^[^ ^133^ ^]^ As the organic solvents are completely removed during the drying process, film rehydration is a biocompatible method and thus, suitable for loading the assemblies with sensitive (bio)molecules. However, hydration times are often long (>24 h) which might affect the functionality of biomolecules, and can lead to the formation of polydisperse and multilamellar vesicles, especially if the rehydration is performed under agitation. To reduce size heterogeneity, which could be disadvantageous for cell mimicking applications, film rehydration‐formed vesicles are usually extruded through a polycarbonate membrane.^[^ ^26^, ^134^, ^135^ ^]^ The resulting small vesicles with low size distribution and high shape homogeneity are often used as artificial organelles.^[^ ^135^, ^136^, ^137^ ^]^ Other methods to reduce size dispersity of film rehydration‐formed vesicles have been developed, such as using patterned surfaces for film formation, which yielded more monodisperse GUVs.^[^ ^138^, ^139^ ^]^ Similar to film rehydration, p‐GUVs can also be generated by depositing polymer films on electrodes and applying alternating electric currents to induce self‐assembly.^[^ ^140^ ^]^ While this method allows formation of more uniformly sized GUVs, other structures such as multilamellar and multivesicular GUVs are also formed. Moreover, control over the size of the vesicles is limited.^[^ ^141^, ^142^ ^]^

Other methods to generate polymersomes in the context of artificial organelles is nanoprecipitation or solvent switch. By adding copolymers dissolved in a water‐miscible solvent to an aqueous buffer containing biomolecules to be encapsulated, self‐assembly can be induced, resulting in mostly nanometer‐sized vesicles. Vesicle size can be controlled via agitation or volume fractions of the phases, resulting in vesicles with narrow size distribution. However, this methods is often associated with low encapsulation efficiency (<30%).^[^ ^143^ ^]^

Polymerization‐induced self‐assembly (PISA) has emerged as a powerful technique for one‐pot assembly of nano‐ and microscale objects from block copolymers.^[^ ^144^ ^]^ PISA involves chain extension of a water‐soluble macroinitiator using water‐miscible monomers that become water‐insoluble at a certain critical degree of polymerization, thereby resulting in self‐assembly. In situ assembly allows PISA to be used for high‐throughput screening of polymeric assemblies.^[^ ^145^, ^146^ ^]^ While PISA is primarily used for nanoscale vesicle generation, it has recently been used also for the generation of GUVs using poly(ethylene glycol)‐*b*‐poly(2‐hydroxypropylmethacrylate) (PEG‐*b*‐PHPMA),^[^ ^147^ ^]^ or poly(glycerol monomethacrylate)‐*b*‐poly(2‐hydroxypropyl methacrylate) (PGMA19‐*b*‐PHPMA25).^[^ ^148^ ^]^ Although PISA is a simple and straightforward method to prepare vesicles with controlled size, surface chemistry, and morphology, it is frequently limited by low encapsulation efficiency and size dispersity when targeting GUVs^[^ ^144^, ^149^ ^]^ as well as by the small selection of suitable monomers. In addition, the vesicles formed are often intrinsically permeable to small molecules, which is incompatible with selective and controllable transport across the membrane.^[^ ^150^ ^]^

An alternative, higher throughput method for creating GUVs is droplet transfer, also known as emulsion centrifugation.^[^ ^45^, ^151^ ^]^ Here, simple water/oil emulsions are generated that are subsequently added to a second, amphiphile‐containing phase layered over an aqueous solution. This is followed by centrifugation, leading to large numbers of vesicles. The size of the vesicles can be controlled by the volume of the internal aqueous phase during the first emulsion step and the emulsification method. Although the uniformity of size is enhanced compared to the aforementioned methods, the resulting vesicles still exhibit considerable size polydispersity.^[^ ^151^, ^152^ ^]^ Yet, a major advantage of this method is the possibility to prepare asymmetric membranes by choosing different amphiphiles for the first emulsion and the second amphiphile‐containing phase.^[^ ^151^ ^]^

The use of microfluidic techniques for the preparation of GUVs has emerged as an ideal method for the control of size, composition, and number of giant vesicles in a high‐throughput process. The first step to obtain GUVs is to prepare water/oil/water (W/O/W) double emulsions. The oil phase can be either organic solvents or oil which is then extracted, resulting in unilamellar vesicles. There are several microfluidic chip designs for the preparation of double emulsions, such as the combination of two T‐branches, two flow‐focusing branches, co‐flow, or flow‐focusing six‐way branches.  This method results in GUVs with extremely high encapsulation efficiencies (approx. 100%) and narrow size distribution.^[^ ^119^, ^153^, ^154^ ^]^ Moreover, through the straightforward manipulation of phase composition and flow rates, both the thickness of the shell, the size, and the composition of the polymer and inner aqueous phases can be rapidly changed. Despite the above advantages, limitations of the microfluidic method include the complex and expensive equipment required for operation and the biocompatibility of the oil phases used. Furthermore, most microfluidic designs are limited to micrometer‐sized channels based on the fabrication methods and operating pressures and therefore mostly employed for the formation of GUVs. By using alternative chip designs such as nanoprecipitation, microfluidic methods may be expanded to produce nanometer‐sized artificial organelles.^[^ ^155^ ^]^

#### 3.2.2 Proteinosomes

For the production of proteinosomes, similar techniques as for the production of polymeric vesicles can be employed. Proteinosomes can be fabricated from proteins that are rendered amphiphilic by the addition of hydrophobic side chains, forming polymer‐protein hybrid amphiphiles.^[^ ^53^, ^54^, ^156^ ^]^

In order to obtain artificial cell mimics, one of the most popular fabrication methods is pickering emulsion self‐assembly.^[^ ^157^ ^]^ This has been demonstrated for temperature‐responsive polymer poly(N‐isopropylacrylamide) (PNIPAAm)‐bovine serum albumin (BSA) amphiphiles using W/O emulsions, where, after oil‐removal and crosslinking, proteinosomes were obtained.^[^ ^53^, ^77^, ^156^ ^]^ While size can be controlled via the initial protein‐polymer concentration, the resulting proteinosomes still exhibit a large size dispersity.^[^ ^35^, ^53^, ^78^ ^]^ In order to better control size and content of proteinosomes, microfluidic methods can be employed and have been shown for the same protein‐polymers to create functional, monodisperse proteinosomes, similar to aforementioned polymer microfluidic methods.^[^ ^54^ ^]^ While proteinosomes can also be produced at nanometer scale,^[^ ^158^, ^159^, ^160^ ^]^ their application as artificial organelles has not been demonstrated so far.

Similarly, actin proteins can also be used to form actinosomes; hollow, micrometer‐sized compartments bounded by a filamentous actin membrane.^[^ ^59^ ^]^ While the architecture is similar to that of a proteinosome, the fabrication method begins with the generation of condensates from polypeptides and nucleoside triphosphates. Actin is then added to the condensate, and localizes to its interface. By polymerizing the actin (with Mg^2+^ and KCl), a hollow membrane‐delimited actinosome can be generated.

#### 3.2.3 Capsosomes

Capsosomes can be produced by sequentially combining polymer capsules and liposomes.^[^ ^62^ ^]^ Incorporating liposomes into polymer capsules prepared by the layer‐by‐layer (LbL) method can improve their limitations such as low stability and structural integrity.^[^ ^161^ ^]^ The preparation starts with the deposition of several polymer layers on a colloidal particle, followed by one or multiple layers of liposomes interspaced by polymeric separation layers. The liposomes may contain different cargoes that determine the functionality of the capsosomes. The LbL assembly involves the combination of oppositely charged polyelectrolytes or functional polymers, which can be linked together through covalent bonding (click chemistry), hydrogen bonding or DNA‐DNA hybridization.^[^ ^63^, ^162^, ^163^, ^164^ ^]^ The layer‐by‐layer assembly is terminated by a final deposition of a protective polymer layer and removal of the core, resulting in hollow capsosomes. This was demonstrated by the alternating deposition of negatively charged or zwitterionic liposomes and/or modified poly(l‐lysine) (PLL) or poly(methacrylic acid) PMA polymer separation layers. The assembly was further capped using a PMA‐layer, and a membrane formed around the capsule by sequential absorption of poly(*N*‐vinylpyrrolidone) (PVP) and PMA.^[^ ^62^, ^64^, ^161^ ^]^

### 3.3 Production of Membrane‐Less Compartments

Coacervates are prepared by liquid–liquid phase separation, forming dense polymer droplets. When coacervates are formed from a self‐condensing ampholyte, they are referred to as simple coacervates. When two oppositely charged polymers are involved, complex coacervates are formed.^[^ ^37^ ^]^ The production of coacervates is achieved through phase‐separation of polymers in solution by changing environmental conditions, such as pH, temperature, or salt concentration. The most popular polymers for the production of coacervates include modified polysaccharides, synthetic polymers, or polypeptides.^[^ ^37^ ^]^

Polysaccharides offer the advantage of possessing many hydroxy functional groups for further modification with charged moieties, of high solubility in water and biocompatibility. Various polysaccharides have been used for the production of coacervates, including amyloses,^[^ ^72^, ^75^, ^165^ ^]^ or dextrans.^[^ ^77^, ^166^, ^167^ ^]^ The advantages of polypeptides in obtaining coacervates include diverse functional side chains and increased biocompatibility based on using natural amino acids as building blocks. Finally, synthetic polymers allow for a tailored design of architecture and functionality of coacervates through their chemical diversity and simple modification. Examples include pH sensitive poly‐l‐lysine/adenosine triphosphate and RNA/spermine coacervates,^[^ ^168^ ^]^ assemblies of poly(diallyldimethylammonium chloride) (PDDA) and carboxymethyl‐dextran (CMDX) into positively charge coacervate micro‐droplets,^[^ ^169^ ^]^ or the coacervation of elastin‐like polypeptide (ELP) and ELP‐*b*‐PEG into coacervates within liposomes through hypertonic shock.^[^ ^170^ ^]^

### 3.4 Production of Artificial Cells: Compartments‐in‐Compartment

Eukaryotic life is characterized by the coexistence of various internal membrane‐bound structures including the nucleus and many organelles. This subcompartmentalization enhances the efficiency of processes by concentrating the necessary components in a confined space. Each organelle acts as a subcellular compartment, contributing to the overall function of the cell.

The bottom–up strategy to generate artificial cells based on polymer components is characterized by several coordinated steps: i) the formation of nano‐assemblies mimicking organelles and equipping them with corresponding active biomolecules, ii) the encapsulation of the organelles inside micrometer sized compartments, and iii) the integration of enzymes, proteins, or cytoskeletal elements that recapitulate necessary cytoplasmic components. The implementation of a compartments‐in‐compartment architecture allows for an increase in complexity and a closer mimic of natural cells. A set of distinct subcompartments is prerequisite for a spatial separation of biochemical reactions, the specific local arrangement of bioactive components, and the bottom–up creation of unique microenvironments, each contributing to the overall cell‐mimetic behavior.

A large variety of nanometer‐sized compartments, such as polymersomes, micelles, nanoparticles, or liposomes have been encapsulated in GUVs to generate simple artificial cells. This was demonstrated by adding polymeric nanoparticles or polymersomes obtained by film rehydration and extrusion (50–200 nm in diameter) to the film rehydration buffer to form GUVs (5–20 µm in diameter).^[^ ^15^, ^34^ ^]^ Similarly, liposomes prepared by film rehydration followed by extrusion, or polystyrene‐*block*‐poly(3‐(isocyano‐lalanyl‐amino‐ethyl)‐thiophene) (PS‐*b*‐PIAT) polymersomes prepared by solvent switch were encapsulated in poly(butadiene)‐*block*‐poly(ethylene oxide) (PBut‐*b*‐PEO) prepared by emulsion centrifugation.^[^ ^14^, ^33^ ^]^ While these techniques are straight‐forward, the encapsulation efficiency is low and control over the number of (co)encapsulated nano‐vesicles is not easily obtained. In bulk methods, the number of encapsulated molecules or compartments is determined not only by the initial concentration but also by method of vesicle fabrication. Through more advanced methods such as double emulsion microfluidics, improved control over the encapsulation process and high encapsulation efficiencies are achieved.^[^ ^79^, ^119^, ^154^ ^]^ However, microfluidic encapsulation of artificial organelles in artificial cells has so far only been demonstrated for lipidic GUVs.^[^ ^20^, ^171^, ^172^ ^]^ A complementary approach to generate artificial cells of more complex architecture is to integrate coacervates designed as models of molecularly crowded, intracellular compartments. For the encapsulation of coacervates in artificial cell compartments, two strategies exist: encapsulation of pre‐formed coacervates in a membrane and the in situ coacervation of precursors within the artificial cell. The first strategy has been demonstrated using poly(ethylene glycol), poly(caprolactone‐gradient‐trimethylene carbonate) and poly(glutamic acid) (PEG‐PCL*g*TMC‐PGlu) on amylose‐based coacervates.^[^ ^72^, ^75^ ^]^ In the latter strategy, coacervation is triggered in situ in artificial cells post assembly by adding a complexation agent, as shown for dextran‐based coacervates in spontaneously assembled proteinosomes.^[^ ^77^, ^78^ ^]^ Other triggers used are pH or osmotic shock in liposomes,^[^ ^168^, ^170^ ^]^ or enzyme‐triggered nucleation in p‐GUVs.^[^ ^42^ ^]^

Although the creation of multi‐compartmental structures with controllable (bio)chemical reagent distribution remains challenging, technological advancements like droplet microfluidics have become invaluable in the controlled fabrication of vesicles and protocells. Microfluidics, in particular, provides the possibility of controlling various aspects of the emulsion formation, such as the shape, size, and composition of the resulting compartments, as well as the rate of formation. The ability to easily exchange of encapsulated bioactive molecules or membrane polymers with high reproducibility can create a modular system for the creation of compartments.^[^ ^42^, ^119^, ^154^, ^170^, ^173^ ^]^ This flexibility allows the creation of artificial cells with highly tunable properties, which are essential for the creation of complex artificial cells.

**Figure 4:**
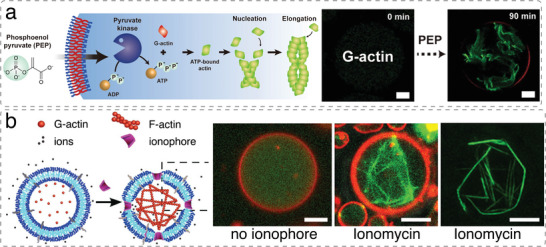
Triggering actin filament formation inside GUVs. a) Actin polymerization inside p‐GUVs induced by phosphoenol pyruvate (PEP). Monomeric (G‐) actin is polymerized in the presence of ATP, resulting in filamentous (F‐) actin. Actin polymerization (green) inside GUV (red) over 90 min upon PEP addition. Scale bars, 20 µm. Reproduced under terms of the CC‐BY license.^[^ ^42^ ^]^ Copyright 2022, Springer Nature. b) Actin polymerization inside p‐GUVs induced by the influx of MgCl_2_. p‐GUVs encapsulating G‐actin and the actin crosslinker filamin were incubated with 150 mm MgCl_2_. In the absence of the ionophore ionomycin, Mg^2+^ is not transported across the p‐GUV membrane (red) and confined actin (green) remains monomeric. When ionomycin was added to the surrounding solution, it reconstituted into the membrane of the p‐GUV and facilitated the entry of Mg^2+^ into the GUV cavity where it triggered actin filament formation and bundling. Projections of actin filaments and filament bundles in p‐GUVs recorded with super‐resolution 3D structured illumination microscopy. Scale bars, 5 µm. Reproduced under terms of the CC‐BY license.^[^ ^34^ ^]^ Copyright 2020, Wiley‐VCH.

Independent of the method used to obtain artificial cells, encapsulation/insertion of bioactive components is fundamental for their functionality. Depending in the task intended, active compounds are encapsulated directly inside artificial cells or inside artificial organelles, and/or in the membranes of both types of compartments. Their in situ activity provides the subcompartments with specific, possibly organelle‐typical functionality. When encapsulated inside the cell‐mimicking compartment, they interact with and/or process products from reactions taking place in subcompartments.^[^ ^14^ ^]^ Enzymes, proteins, and nucleic acids are used as bioactive components as they play a role in catalysis, signal processing, and gene expression, respectively.^[^ ^119^, ^174^, ^175^, ^176^, ^177^ ^]^ In addition, molecular building blocks can be encapsulated within artificial cells and their supramolecular assembly triggered in situ using external stimuli, which bestows artificial cells with dynamic behavior and allows them to respond to changes in environmental conditions. For example, monomeric actin was induced to assemble actin bundles in p‐GUVs in the presence of actin‐crosslinking proteins and salt (**Figure** **4**).^[^ ^34^, ^42^ ^]^

For coacervate‐based artificial cells, the bioactive components are usually crosslinked to the coacervate or become an integrate part of the coacervate during phase separation.^[^ ^75^, ^77^ ^]^

A unique set of conditions and operations is tied to the integration of biomolecules within the membrane of synthetic compartments. The biomolecules to be incorporated within the membrane are usually hydrophobic or amphiphilic and serve to facilitate the traversing of molecules across the membrane (pore‐forming peptides and proteins, ionophores) or participate in specific reactions and interfacial interactions (enzymes or proteins). The simplest form of membrane transporter, the ionophores, mediates the influx of externally added ions. This was demonstrated using ionomycin, which allow the selective transport of Mg^2+^ and Ca^2+^ ions across an otherwise impermeable PDMS‐*b*‐PMOXA membrane. Interestingly, a difference was observed depending on whether the ionophore was added from the outside or from within the p‐GUV, suggesting that the rate of passive diffusion and the local ion concentration mattered.^[^ ^15^, ^34^, ^48^ ^]^

The insertion of pore‐forming proteins and peptides into synthetic membranes enables the diffusion of a broader range of molecules to and from the compartment interior albeit with limitations regarding molecular weight, shape and charge. The incorporation of membrane proteins also allows for detailed biophysical studies of the channel properties. For example, the pore‐forming peptide melittin was used to permeabilize synthetic membranes assembled from PDMS‐*b*‐PMOXA di‐ and triblock copolymers, obtaining pores that allow passage of molecules up to 4 kDa.^[^ ^88^ ^]^ In contrast to pores that dynamically assemble within the membrane, preformed membrane pores can also be used for membrane permeabilization. A prominent example is outer membrane protein F (OmpF) from *Escherichia coli* which was reconstituted in polymersomes and in microfluidic artificial cells made from PDMS‐*b*‐PMOXA diblock copolymers.^[^ ^119^, ^121^, ^124^ ^]^ Similarly, the toxin α‐hemolysin from *Staphylococcus aureus* forms transmembrane pores upon contact with membranes, and is widely used for the permeabilization of p‐GUVs.^[^ ^120^, ^178^ ^]^ A step forward was achieved by insertion of chemically or genetically engineered membrane channels into compartments membrane serving to support a stimuli‐responsive permeability.^[^ ^179^, ^180^ ^]^ While the permeabilization via natural membrane pores is wide‐spread, synthetic pores are emerging as an alternative as they can be tailored to specific membranes and predefined void sizes. For example, DNA origami nanopores have been successfully inserted into p‐GUVs.^[^ ^122^ ^]^

The membranes of artificial cells and organelles can also be decorated at their external interface with biomolecules in order to support their clustering. Such surface modifications enable mimicking the spatial arrangement of natural organelles, as well as interactions among and between artificial cells and bridging with biological cells.^[^ ^113^ ^]^ While clustering of artificial organelles has been achieved and used for medical applications,^[^ ^113^ ^]^ clustering of artificial cells has so far only been explored for lipidic membranes.^[^ ^44^, ^181^, ^182^ ^]^

## 4 Mimicking Functionality of Organelles and Cells: Reactions inside Synthetic Organelles and Cells

Compartmentalization plays a vital role in organizing the complex network of reactions in the eukaryotic cell into specialized functional spaces, enabling their spatial and temporal control. Most enzymatic reactions in the cell take place under conditions of molecular crowding either in the cytosol, or in volume‐confined compartments such as membrane‐bound organelles, both conditions having major effects on the kinetics of a reaction. For many reactions that take place in specific membrane‐bound organelles, substrate and product molecules have to get across the membrane, while the enzyme is confined and sequestered from the surroundings. Therefore, compartment boundaries need to be selectively permeable. In contrast, membrane‐less organelles promote biochemical reactions by simultaneously concentrating substrates and enzymes, and/or suppressing the activity of these sequestered factors elsewhere in the cell.^[^ ^183^ ^]^

In cases where cells are deficient of certain enzymes or lacking them altogether, artificial organelles could serve to restore or add enzyme activity. Apart from exploiting naturally occurring enzymatic reactions or supporting their activity, artificial organelles once internalized can expand the cell's repertoire of enzymatic functionality, for example by catalyzing the synthesis of drugs from prodrugs.^[^ ^184^, ^185^, ^186^ ^]^

### 4.1 Reactions in Synthetic Organelles

In their simplest form, artificial organelles confine one type of enzyme by a selectively permeable polymer membrane that allows for the exchange of enzyme substrates and products with the surrounding. A more complex approach is to co‐encapsulate different active biomolecules that carry out a cascade reaction inside a single nanocompartment. An even higher hierarchical organization is achieved by sequestering the enzymes in different reaction compartments that are then either mixed at specific concentrations to allow them to work in tandem or arranged in spatially defined clusters.

#### 4.1.1 Reactions in Simple Artificial Organelles

Reactions inside nanocompartments by encapsulation of single enzymes were first proposed as nanoreactors acting in bulk,^[^ ^121^ ^]^ with the aim to explore whether biomolecules preserve their activity inside polymersomes. In order to develop artificial organelles for future medical applications, simple catalytic compartments encapsulating one type of enzyme have been introduced into cells to produce desired compounds or detoxify harmful molecules.^[^ ^134^, ^175^, ^179^, ^187^, ^188^, ^189^, ^190^ ^]^

An early example of catalytic polymersomes showing activity inside cells are PMOXA‐*b*‐PDMS‐*b*‐PMOXA polymersomes loaded with trypsin and decorated with the oligonucleotide polyG to specifically target macrophages.^[^ ^188^ ^]^ Internalized polymersomes were stable and maintained biochemical functionality over 48 h. Likewise, polymersomes made from polystyrene‐*b*‐poly[l‐isocyanoalanine(2‐thiophen‐3‐yl‐ethyl)amide] (PS‐*b*‐PIAT) and 10% polystyrene‐*b*‐poly(ethylene glycol) that were loaded with horse radish peroxidase (HRP) demonstrated catalytic activity in HeLa cells.^[^ ^190^ ^]^ To promote cellular uptake, a cell‐penetrating TAT peptide was conjugated to the surface of these polymersomes.

**Figure 5:**
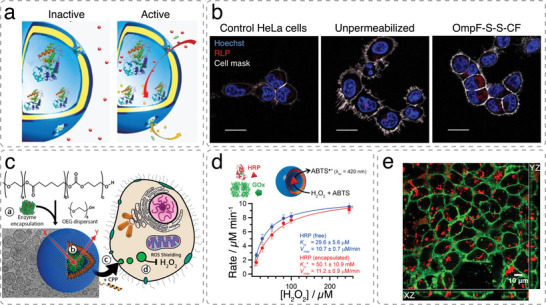
Catalytic nanocompartments encapsulating one type of enzyme demonstrate their activity inside cells. Permeability toward substrates and products is achieved through a,b) membrane proteins or c–e) by using intrinsically permeable polymer membranes. a) Schematic representation of modified OmpF acting as a gate in catalytic polymersome nanocompartments. b) Cellular uptake and intracellular activation of fluorescently labelled HRP‐loaded polymersomes producing resorufin‐like product (RLP; red). Scale bar 20 µm. Reproduced with permission.^[^ ^179^ ^]^ Copyright 2018, Springer Nature. c) Preparation of biodegradable enzyme‐loaded PEG‐*b*‐PCL*g*TMC polymersomes via self‐assembly and model of function as artificial organelles inside cells. Semipermeable enzyme‐loaded nanocompartments were decorated with a cell‐penetrating peptide (CPP) to promote cellular integration as antioxidant organelles. d) Michaelis–Menten kinetics plot of HRP‐loaded artificial organelles and free enzyme. Encapsulation within polymersomes leads to an increase in *K*_M_ due to the diffusion barrier. e) Uptake of polymersomes in HEK293T cells. Confocal microscopy images showing the subcellular distribution of TAT‐polymersomes encapsulating AF647BSA (red) after 24 h of incubation at a concentration of 0.4 mg mL^−1^. Plasma membranes are stained with CellMask green. Reproduced with permission under terms of the CC‐BY‐NC‐ND license.^[^ ^83^ ^]^ Copyright 2018, American Chemical Society.

In order to use artificial organelles as cellular implants supporting the functionality of the cells, it is necessary to demonstrate their activity in vivo. One challenge in the design and development of artificial organelles for in vivo application is the need to specifically trigger their functionality inside the cells. Redox‐responsive PMOXA‐*b*‐PDMS‐*b*‐PMOXA polymersomes encapsulating HRP as a model enzyme were developed to be catalytically active in the presence of intracellular glutathione.^[^ ^179^ ^]^ The redox response was achieved by integrating genetically modified OmpF into the polymer membrane, which served as a stimuli‐responsive “gate” for the substrates of the enzymatic reaction. Intracellular glutathione triggered the release of a molecular cap from the engineered OmpF pores such that the substrate entered the compartment and was enzymatically converted to a detectable product (**Figure** **5**a). Stimuli‐responsive activity of these artificial organelles was shown in HeLa cells (Figure 5b) and in zebrafish embryos, demonstrating the feasibility of using artificial organelles as cellular implants in living organisms.

**Figure 6:**
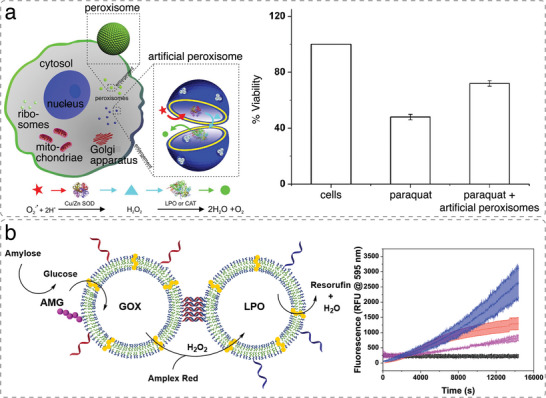
Cascade reactions between enzymes co‐encapsulated within the same compartment and enzymes in clustered compartments. a) Schematic illustrating artificial peroxisome based on enzymatic cascade for the detoxification of superoxide radicals and related H_2_O_2_ inside cells (upper panel). The artificial organelle is based on the simultaneous encapsulation of a set of antioxidant enzymes in a polymersome, with a membrane equipped with channel proteins. Flow cytometry analysis demonstrates the protective effect of artificial peroxisomes (PQ‐Aps) when cells (viability 100%) are treated with paraquat (PQ) (down). Reproduced with permission.^[^ ^82^ ^]^ Copyright 2013, American Chemical Society. b) Schematic representation of the AMG–GOx–LPO cascade between two clustered polymersomes, tethered via complementary ssDNA (upper panel). Enzymatic activity of AMG(GOx)–LPO clusters (blue), unclustered compartments (red), (GOx)–LPO clusters with AMG in the solution (magenta) and Amplex Red autoxidation (black) (down). Reproduced with permission under terms of the CC‐BY‐NC license.^[^ ^81^ ^]^ Copyright 2021, Royal Society of Chemistry.

The next step toward mimicking biochemical pathways is to implement cascade reactions inside a single compartment. The proximity of interacting enzymes improves the reaction kinetics, resulting in a high reaction efficiency due to shorter diffusion times and a reduced likelihood of side reactions. Apart from residing in the lumen of the compartment, enzymes can be integrated in the membrane, or tethered on the surface, enabling cascade reactions with different spatial positioning.^[^ ^191^, ^192^ ^]^ Several examples exist where enzymes participating in the cascade were co‐encapsulated in a single nanocompartment, although the loading efficiency has been rather low (**Figure** **6**a).^[^ ^82^, ^137^, ^193^, ^194^, ^195^ ^]^

Complexity is further increased by cascade reactions over segregated reactions spaces. A cascade reaction involving uricase and HRP has been explored between nanocompartments to understand the conditions and distance between segregated reaction spaces that are necessary to optimize the cascade and mimic bioconditions in cell reactions. Furthermore, an efficient multi‐step reaction involving different polymer compartments requires controlled mass transport of reactants and products across the compartment boundary. To accomplish this, intrinsically permeable polymer membranes have been used to establish two‐step,^[^ ^197^, ^198^ ^]^ and three‐step cascade reactions between enzymes encapsulated in the lumen of polymersomes.^[^ ^199^ ^]^ Alternatively, exchange of substrates and products between compartments was enabled through the insertion of OmpF in PMOXA_6_‐*b*‐PDMS_44_‐*b*‐PMOXA_6_ polymersomes.^[^ ^196^, ^200^ ^]^ Uricase (UOX) and HRP were loaded in separate polymersomes to support the non‐native UOX‐HRP cascade between the two compartments, where H_2_O_2_ produced by UOX‐catalyzed oxidation of uric acid in compartment one is used by HRP in compartment two to catalyze conversion of a substrate to a fluorescent product.^[^ ^196^ ^]^ This enzymatic cascade offers an effective approach for detoxification of harmful uric acid and prevention of H_2_O_2_ accumulation, showing a potential for treating gout and mitigating oxidative stress. Cascade reaction efficiency was evaluated at different concentrations in order to better understand the effect of intercompartment distance on communication. A balance between the protective function of polymersomes and the permeation of small molecules is necessary to ensure efficient cascade reactions. Tandem catalytic nanocompartments remained active in biofluids and upon incubation with kidney‐derived HEK293T cells.

Although the clustering of reaction compartments provides the means to improve the efficiency, so far there are only few examples of reactions in linked nanocompartments.^[^ ^81^, ^174^, ^201^ ^]^ In a recent example, PDMS‐*b*‐PMOXA polymersomes with separately encapsulated GOx and lactoperoxidase (LPO) enzymes were tethered together by DNA hybridization of complementary single‐stranded DNA on the surface of different polymersomes.^[^ ^81^ ^]^ This system demonstrated an improved GOx‐LPO cascade efficiency compared to combined, non‐tethered polymersomes encapsulating either GOx or LPO. To add more complexity to the enzymatic cascade, amyloglucosidase (AMG) was coupled to the surface of GOx‐nanocompartments, enabling AMG‐GOx‐LPO cascade reaction (Figure 6b). Surface‐attached AMG catalyzed the hydrolysis of bulky amylose to glucose, that can then enter the permeabilized polymersome where it is converted by GOx to gluconic acid and H_2_O_2_. The latter is used by LPO to oxidize Amplex red to resorufin. Complementary single‐strand DNA on the surface of polymersomes not only allows to control the inter‐polymersome distance but provide the means to cluster different compartments in a geometry that is conducive to the cascade reactions.

#### 4.1.2 Nanocompartments with Bioorthogonal Functionality

Nanosized compartments that inside natural cells either support, correct or replace endogenous processes, or introduce new, orthogonal functionalities are considered artificial organelles. Examples of their functionalities are supporting cellular detoxication or converting nontoxic prodrugs into cytotoxic drugs in targeted cells. Artificial organelles producing drugs in situ, often referred to as therapeutic nanofactories, are mostly employed in anticancer therapy.^[^ ^202^, ^203^ ^]^ In one such example, artificial organelles produced by self‐assembly of carbohydrate‐conjugated polymers were loaded with β‐galactosidase (β‐gal) and DOXgal prodrug, and decorated with a TAT peptide to enhance cellular uptake. β‐gal catalyzed hydrolysis of DOXgal into active doxorubicin (DOX) upon cellular uptake of the polymersomes was shown to inhibit the growth of several cancer cell lines as well as the growth of tumors in a mouse model.^[^ ^186^ ^]^ Biocatalytic polymeric nanofactories were also used for combined cancer therapy.^[^ ^204^ ^]^ GOx‐loaded polymersomes were assembled from polymers with the drug camptothecin (CPT) conjugated to their side chain by an oxalate ester linker. The piperidine moieties of these polymersomes rendered them pH‐responsive. At the slightly acidic pH of the tumor microenvironment, piperidine groups were protonated which increased the permeability of the polymer membrane, allowing for the entry of glucose into the polymersome. Activated in this way, GOx produced high levels of H_2_O_2_ which on one hand cleaved the oxalate ester linker to release CPT and at the same time increased the oxidative stress in the tumor tissue. The synergistic effect of oxidative stress and chemotherapy was corroborated in a mouse tumor model, where the tumors were almost completely eliminated at the end of treatment with nanofactories.

#### 4.1.3 Reconstructing Natural Organelles

Of particular interest are artificial organelles designed to mimic reactions that are typical for natural organelles, such as peroxisomes, lysosomes and mitochondria. By mimicking mitochondria and chloroplasts, which are core structures of energy conversion in living cells, artificial organelles can serve as energy supply modules.^[^ ^205^ ^]^ A nanocompartment for ATP regeneration was developed by combining the ATP synthase with the proton pump bo_3_ oxidase in PDMS‐*g*‐PEO polymersomes and PDMS‐*g*‐PEO/phosphatidycholine hybrid vesicles.^[^ ^206^ ^]^ The compartments took on mitochondrial features by selective phosphorylation of ADP to create the energy‐rich ATP. These artificial organelles were able to continuously generate ATP for several hours. When artificial mitochondria were adequately supplied with cofactors, physiological levels (0.5–5 mm) of ATP were produced. However, ATP synthesis rates were lower in polymersomes compared to hybrid nanovesicles, and even lower than production rates obtained with liposomes.

Lysosomes are membrane‐bound organelles with an acidic interior (pH ≈ 4.5–5.0), which contains a variety of digesting enzymes that are able to break down many biomolecules. Artificial lysosomes capable of digesting biological material in different environments were fabricated by encapsulating trypsin in the lumen of pH‐responsive poly(ethylene glycol)‐b‐poly(2‐(*N*,*N’*‐dimethylamino)ethyl methacrylate)‐*co*‐3,4‐dimethylmaleic imidobutyl methacrylate) (PEG‐*b*‐p(DEAEMA‐*co*‐DMIBMA)) polymersomes.^[^ ^207^ ^]^ At acidic pH, protonation of the membrane‐forming polymer induced membrane swelling accompanied by an increase in permeability which allowed for the access of biomolecule substrates below a molecular weight cut‐off of around 40 kDa. Accordingly, the artificial organelle showed a pH‐dependent lysosome‐like digestion by trypsin of myoglobin (16.7 kDa) but not of HRP (44 kDa). Digestion was also promoted by a partial trypsin integration into the polymer membrane.

Peroxisomes play an essential role in regulating reactive oxygen species (ROS) such as superoxide radicals and H_2_O_2_ via diverse oxidative enzymes. Artificial organelles encapsulating ROS detoxifying enzymes, such as superoxide dismutase (SOD), catalase (CAT) and lactoperoxidase (LPO), were designed to mimic peroxisomes (Figure 6a).^[^ ^82^, ^137^ ^]^ Combinations of SOD and CAT or LPO carry out a cascade reaction that converts superoxide radicals and H_2_O_2_ to molecular oxygen and water. Thus, SOD/LPO and SOD/CAT were co‐encapsulated in PMOXA‐*b*‐PDMS‐*b*‐PMOXA polymersomes permeabilized by OmpF.^[^ ^82^ ^]^ The resulting artificial peroxisomes were taken up by cancer cells which then demonstrated enhanced survival when exposed to oxidative stress. In a simpler scenario, only one antioxidant enzyme was encapsulated inside polymersomes to detoxify ROS. Various reactive oxygen and nitrogen reactive species were detoxified by encapsulated SOD,^[^ ^134^, ^175^, ^208^ ^]^ and CAT.^[^ ^83^, ^209^ ^]^ For example, intrinsically permeable PEG‐*b*‐PCL*g*TMC polymersomes loaded with CAT were able to degrade exogenous H_2_O_2_ in human embryonic kidney cells and fibroblasts (Figure 5c–e).^[^ ^83^ ^]^ Equipping the surface of these simple one enzyme‐based artificial peroxisomes with a cell‐penetrating peptide (TAT) promoted their cellular uptake which, in turn, enhanced their ROS shielding effect. The therapeutic potential of antioxidative artificial organelles was supported by several in vivo studies using SOD‐loaded polymersomes assembled from a poly(ethylene glycol)‐*b*‐polybutadiene (PEG‐*b*‐PBD)/poly(ethylene glycol)‐*b*‐poly(propylene oxide) (PEG‐*b*‐PPO) mix.^[^ ^134^, ^175^, ^208^ ^]^ Corresponding antioxidant nanocompartments targeting the synovium were applied to mice suffering from osteoarthritis, and by minimizing oxidative damage verified their therapeutic capacity. Antioxidant enzyme therapy with aforementioned SOD‐loaded polymersomes was also used to preserve cardiac function in rats after myocardial injury.^[^ ^208^ ^]^ Moreover, a similar system was used to mitigate neuropathic pain after nerve root compression in rats.^[^ ^134^ ^]^

Melanosomes, organelles present in melanocytes, produce and store melanin pigments that protect the skin from UV damage. Melanosome mimics were fabricated by encapsulating the melanogenic enzyme tyrosinase together with l‐DOPA/dopamine, precursors of melanin and polydopamine, in PDMS‐*b*‐PMOXA polymersomes under formation conditions that promote melanogenesis within the cavity.^[^ ^210^ ^]^ The potential of artificial melanosomes for photoprotection was indicated by keratinocytes which, after UV‐irradiation, showed enhanced viability compared to control cells.

### 4.2 Multicompartment Reactions in Artificial Cells

Various multicompartmentalized systems were used to realize simple cascade reactions and achieve intracellular communication in p‐GUVs,^[^ ^14^, ^211^, ^212^, ^213^ ^]^ coacervates,^[^ ^60^, ^66^, ^76^, ^80^, ^161^, ^214^, ^215^ ^]^ and proteinosomes.^[^ ^35^, ^77^, ^216^ ^]^

**Figure 7:**
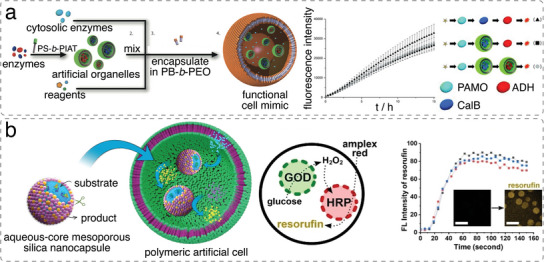
Cascade reactions in multicompartment systems based on p‐GUVs. a) Different enzymes were encapsulated within PS‐*b*‐PIAT polymersomes, mixed with cytosolic enzyme and substrates and encapsulated in PB‐*b*‐PEO p‐GUVs to fabricate polymersome‐in‐p‐GUV artificial cells hosting multicompartment catalysis. Different cascade reactions of increased complexity were investigated and monitored with fluorescent spectroscopy. Reproduced with permission.^[^ ^45^ ^]^ Copyright 2014, Wiley‐VCH. b) Enzyme‐loaded silica nanocapsules were used as organelles to construct a catalytic p‐GUV multicompartment system. The GOx‐HRP cascade in the microreactor was followed using confocal laser scanning microscopy to measure the generation of fluorescent resorufin product. Reproduced with permission.^[^ ^211^ ^]^ Copyright 2022, Wiley‐VCH.

Different artificial organelles were successfully encapsulated in p‐GUVs to mimic the hierarchically compartmentalized reactions in eukaryotic cells. The first polymersomes‐in‐p‐GUV catalytic system able to execute a 3‐step cascade reaction was developed by encapsulating enzyme‐loaded PS‐*b*‐PIAT polymersomes within PB‐*b*‐PEO p‐GUVs.^[^ ^14^ ^]^ The enzymes alcohol dehydrogenase and CalB or alcalase were encapsulated in different PS‐*b*‐PIAT polymersomes, while phenylacetone monooxygenase (PAMO) was used as a cytosolic enzyme (**Figure** **7**a). Communication between enzymes confined in polymersomes and those in the surrounding medium was also shown with OmpF‐permeabilized HRP‐loaded PMOXA‐*b*‐PDMS‐*b*‐PMOXA polymersomes that were encapsulated together with GOx into larger PS‐*b*‐PIAT polymersomes.^[^ ^213^ ^]^

Silica core‐shell nanoparticles were also incorporated as subcompartments into p‐GUVs (Figure 7b).^[^ ^211^, ^212^ ^]^ HRP and GOx as model enzymes were encapsulated in these semipermeable silica nanocapsules of high colloidal stability. The in situ encapsulation approach is also suitable for the co‐encapsulation of enzymes in well‐defined compositions. Enzyme‐loaded core–shell particles served as functional nanocompartments when integrated into PB‐*b*‐PEO GUVs, the resulting compartments‐in‐compartments supporting the GOx‐HRP cascade.

Coacervates stabilized with self‐assembled polymer membrane were used to fabricate active multicompartments, as for example cell‐sized amylose‐based coacervate microdroplets enclosed by a PEG‐PLC*g*TMC‐Pglu membrane.^[^ ^76^ ^]^ Terpolymer was successfully anchored through the electrostatic interaction of anionic poly(glutamic acid) and positively charged coacervate. To form subcompartmentalized artificial cells, negatively charged polymersomes containing either separately or co‐encapsulated GOx and HRP, were incorporated into the coacervates, which were then covered by the membrane of the terpolymer. The GOx‐HRP cascade reaction took place within the polymersome‐based organelles with improved kinetics when both enzymes were located in the same polymersomes compared to separately encapsulated enzymes. The coacervate served as crowded environment of a model cytosol while the polymer shell played the role of membrane boundary, making it a suitable platform for studying reactions in a cell‐like environment.

The first catalytic capsosomes were constructed by sandwiching β‐lactamase‐loaded liposomes between a poly(l‐lysine) (PLLc) precursor layer and a poly(methacrylic acid)‐*co*‐(cholesteryl methacrylate) (PMAc) capping layer, demonstrating that the enzyme remains active inside this multi‐compartment system.^[^ ^66^ ^]^ By loading liposomes with different enzymes, an increased complexity was achieved in capsosomes. Some of the examples include two single‐enzyme conversions in parallel using HRP and trypsin,^[^ ^214^ ^]^ bi‐enzymatic cascades such as GOx‐HRP,^[^ ^161^ ^]^ and uricase‐HRP cascade, in parallel with the ascorbate oxidase single‐enzyme conversion.^[^ ^60^ ^]^

Moreover, two different classes of subcompartments, liposomes and hydrogel polymeric capsules, were introduced in capsosomes.^[^ ^215^ ^]^ Liposomal and polymer capsule layers were separated by a poly(*N*‐vinyl pyrrolidone‐*b*‐(cholesteryl acrylate) (PVPc) layer that was destabilized by an increase of pH, enabling the generation of “free‐floating” liposomes and thus a control over the spatial positioning of the subcompartments. “Free‐floating” lipid subcompartments were significantly better mimics of the arrangement of organelles inside eukaryotic cells compared to the immobile layer of subcompartments. Moreover, hydrogel subcompartments were cross‐linked with disulfide bonds, making them degradable in the presence of reduction agents. The addition of glutathione lead to the disassembly of only polymeric subcompartments, whereas β‐lactamase loaded liposomes remained stable. β‐lactamase encapsulated within them maintained its catalytic activity 24 h after the selective degradation of polymer subcompartments, which was demonstrated by the conversion of the yellow nitrocefin into its red hydrolyzed product.

**Figure 8:**
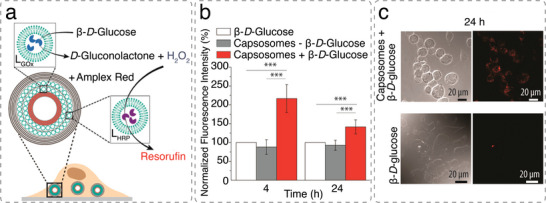
Intracellular activity of capsosomes containing enzyme‐loaded liposomes. a) β‐D‐Glucose can permeate through the liposomes membrane and be converted by GOx into d‐gluconolactone and H_2_O_2_. H_2_O_2_ reacts with Amplex Red reagent in the presence of HRP to generate the fluorescent resorufin. b) Fluorescence intensity of the enzymatic cascade conversion monitored by fluorescence spectroscopy at 4 and 24 h. Macrophages had been incubated with the capsosomes prior to the addition of substrates for 4 h to allow cell internalization (*n*  = 3, ***p < 0.001). c) Differential interference contrast (DIC, left) and confocal laser scanning (CLSM, right) microscopy images showing the enzymatic cascade conversion of β‐d‐glucose into the red fluorescent product resorufin by capsosomes loaded with GOx and HRP after and 24 h. Control images, in which the substrates were incubated with the macrophages in the absence of capsosomes, are shown at the bottom. Reprinted with permission.^[^ ^161^ ^]^ Copyright 2017, American Chemical Society.

Furthermore, capsosomes were shown to be catalytically active inside natural cells, specifically in a macrophage cell line where they were internalized without affecting cell viability (**Figure** **8**).^[^ ^161^, ^214^ ^]^ Apart from adding liposomes with enzyme cargo (GOx and HRP, trypsin, and HRP) during the assembly process, gold nanoclusters (AuNCs) were also entrapped, making it possible of monitor the cellular uptake of the system by AuNC fluorescence. This system represents a first step toward theranostic applications, with AuNCs serving as the imaging modality and encapsulated enzymes as a model therapeutic. In addition, capsosomes with tyrosinase encapsulated in liposomal subunits were able to inhibit melanoma cell growth by depleting l‐tyrosine, which is crucial for tumor growth. These systems were able to maintain their catalytic activity in the vicinity of melanoma cells and under conditions of intratumor shear stress.^[^ ^80^ ^]^

To further advance the concept of cell‐mimics, the complexity of compartments‐in‐compartment was increased by co‐encapsulation of up to five enzymes involved in two distinct enzymatic pathways: glutamate dehydrogenase and glutathione reductase, using NADP^+^/NADPH as a self‐renewable co‐factor, and an enzymatic cascade combining the enzymes β‐gal, GOx, and CAT, using lactose as substrate and generating water and oxygen.^[^ ^217^ ^]^ The compartments‐in‐compartment system was assembled by combining polymer layers and enzyme‐loaded liposomes as subcompartments, which physically separates the two encapsulated enzymatic pathways. These capsosomes carried out both cascade reactions simultaneously.

Proteinosomes were also developed to mimic cells, as for example pancreatic cells.^[^ ^35^ ^]^ A hierarchical polymersomes‐in‐proteinosome micro‐sized system was constructed by incorporating pH‐responsive polymersomes loaded with PEGylated insulin as artificial organelles and GOx into protein hydrogel‐filled proteinosomes based on BSA‐NH_2_/PNIPAAm. In the presence of glucose, the pH of the microcompartment decreased due to the enzymatic activity of GOx, leading to the swelling of the polymersomes which, in turn, allowed the release of PEGylated insulin. The protein hydrogel inside the proteinosomes was composed of cationized BSA and aldehyde‐dextran and acted as a cytoskeleton to promote the even distribution of polymersomes based on charge interactions and to enhance the mechanical stability of the cell‐like multicompartmental structure. A similar concept was used to construct artificial cells capable of glucose detection.^[^ ^216^ ^]^ pH‐responsive polymersomes encapsulating alkaline phosphatase (ALP) were co‐loaded with GOx and CAT inside proteinosomes. ALP within artificial organelles catalyzed the hydrolysis of fluorescein diphosphate (FDP) to a fluorescent dye. GOx‐catalyzed oxidation of glucose in the lumen of proteinosome produced H_2_O_2_, which was converted to water and oxygen by CAT, and gluconolactone that when hydrolyzed to gluconic acid, lowered the pH. Change in the pH modulated the activity of ALP inside subcompartments, making them adaptive artificial organelles for the detection of extracellular glucose.

**Figure 9:**
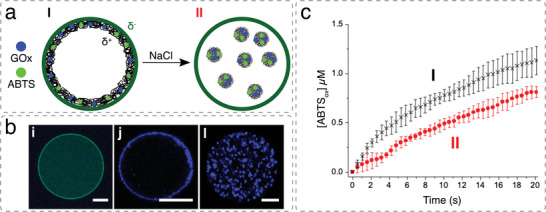
Coacervate‐in‐proteinosome artificial cells hosting GOx‐HRP cascade. HRP is integrated in the proteinosome membrane (green circle) while GOx is associated with the ATP/PDDA coacervate phase. a) Electrostatically induced spatial positioning and relocation of entrapped coacervate and GOx from arrangement I (thin coacervate shell located directly on the HRP‐active membrane) to arrangement II (entrapped dispersion of coacervate microdroplets. b) Confocal fluorescence microscopy images of a single proteinosome showing i) green fluorescent HRP membrane with PDDA and GOx encapsulated inside, j) coacervate phase forming a thin layer under the proteinosome membrane after the addition of ATP, recorded as blue‐filtered image, and l) relocation of the GOx/coacervate phase into droplets away from the HRP‐containing membrane after the addition of NaCl. c) Increase in [ABTS_ox_] over time for the GOx‐HRP cascade reactions in coacervate‐in‐proteinosome artificial cells organized in arrangements I or II. Reproduced with permission.^[^ ^77^ ^]^ Copyright 2019, Wiley‐VCH.

Coacervates were also introduced as subcompartments in proteinosomes, forming coacervate‐in‐proteinosome artificial cells.^[^ ^77^ ^]^ Coacervate droplets (between 500 nm and 1.5 µm) were formed from polyelectrolyte poly(diallyldimethyl‐ammonium chloride) (PDDA) and ATP, and loaded with GOx to produce positively charged GOx‐containing ATP/PDDA (**Figure** **9**a). The coacervate phase was hosted inside a negatively charged BSA‐NH_2_/PNIPAAm proteinosome outer membrane. HRP‐NH_2_/PNIPAAm was added during assembly and crosslinked, producing an HRP‐containing proteinosome membrane. The coacervate phase has been positioned as a thin layer under the negatively charged proteinosome membrane or within the aqueous lumen, while the configuration of entrapped coacervates was controlled by electrostatic interactions between the positively charged coacervate and surrounding negatively charged proteinosome (Figure 9b). Consequently, there were two possible modes of glucose‐stimulated GOx‐HRP cascade reactions between membrane‐incorporated HRP and coacervate‐sequestered GOx that is either positioned as a thin layer or dispersed as microdroplets within the aqueous lumen (Figure 9c). Cascade modes were regulated by tuning the electrostatic interactions between entrapped coacervate phase and the proteinosome membrane. A change of spatial configuration was induced by the addition of NaCl which caused the disassembly of thin coacervate layer and the formation of coacervate droplets in the lumen (Figure 9a). This way, coacervation was achieved using a membrane‐permeable trigger, circumventing the need for temperature or osmotic‐pressure to induce the phase transformation.

Although significant progress has been made in fabricating multicompartment architectures, their functionality remains relatively undeveloped. In most cases, a GOx‐HRP or similar simple model cascade reactions were used to demonstrate the biocatalytic potential of artificial cells. While essential as early stage research, such bi‐enzymatic reactions inside artificial cells represent proof of concept and exhibit only limited resemblance to biologically relevant reactions, which are yet to be mimicked in a more complex way.

## 5 Harnessing the Ability for Adaptive Behavior

In biological cells, the ability to sense changes in the environment and subsequently process this information is prerequisite for the precise regulation of complex biochemical reaction networks in space and time, and ultimately for survival. Importantly, continuous communication with the environment and between cells is key to coordinate cellular responses in ensembles of cells such that higher‐order collective behavior can be achieved. Therefore, signal sensing, information processing, and communication with other cells, resulting in adaptive changes are basic evolution requirements. In fact, cell signaling is thought to have evolved before multicellularity.^[^ ^218^ ^]^

### 5.1 Outside‐in Signaling in Artificial Cells

**Figure 10:**
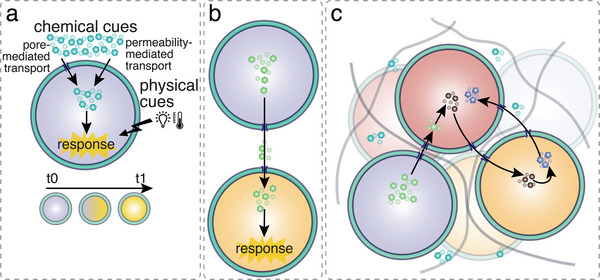
Signaling pathways in artificial cells. a) Outside‐in signaling. Chemical and physical signals from the environment trigger activity inside the artificial cell. b) Cell–cell communication through diffusive transmission and reception of chemical cues. c) Intercellular signaling networks for coordinated activity in ensembles of artificial cells.

Cells can sense a sheer limitless variety of physical and chemical signals including temperature, light, mechanical cues, pH, salt, small molecules, and peptides (**Figure** **10**). They employ different mechanisms including the uptake and secretion of molecules, receptor‐mediated signaling across cell boundaries, and mechanotransduction for transmitting these signals across the membrane which provide the basis for inter‐ and intracellular communication. As with natural cells, the ability to respond to chemical or physical signals is crucial for the adaptation of artificial cells to a dynamic environment. Bottom–up engineering enables artificial cells to acquire corresponding sensitivities such that desired activities can be specifically triggered by providing appropriate signals (Figure 10a). Examples include the heat‐triggered content release functionality described for l‐GUVs,^[^ ^219^ ^]^ histamine‐induced riboswitches that activate protein expression in or cargo release from lipid vesicles,^[^ ^220^ ^]^ and DTT‐triggered enzyme activity inside p‐GUVs with a concomitant release of ionophores from encapsulated nanoassemblies.^[^ ^15^ ^]^

**Figure 11:**
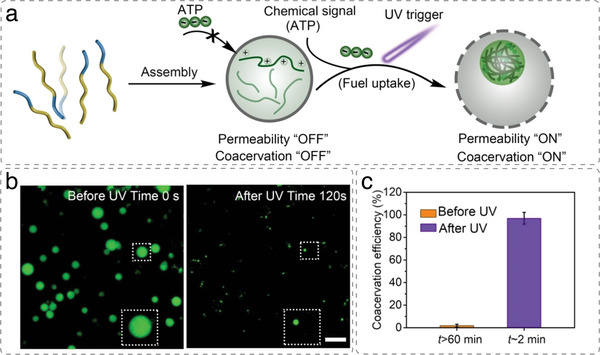
Light‐triggered formation of sub‐compartments inside SP‐GPs. a) Cartoon illustration of the light‐triggered formation of coacervate‐in‐polymersome. The external chemical signal (ATP) cannot initially cross the vesicle membrane. Upon UV irradiation, the enhanced SP‐GP permeability enables the crossing of ATP into the aqueous lumen of the vesicles, inducing the condensation of poly‐l‐lysine (PLL) and the formation of internal coacervate droplets. b) Confocal microscopy images of fluorescently labeled PLL present inside SP‐GPs. The external solution contained 1 mg mL^−1^ ATP. Images show PLL before UV and PLL coacervation 120 s after UV irradiation. Scale bar: 50 µm. c) Relative coacervation efficiency with and without UV irradiation (20 s UV irradiation). Reproduced with permission under terms of the CC‐BY‐NC license.^[^ ^225^ ^]^ Copyright 2022, Wiley‐VCH.

Responsiveness to a pH change in the environment has been reported for a number of polymeric and hybrid GUVs. For example, pH‐responsive hybrid vesicles were formulated with a poly(dimethylsiloxane)‐*b*‐poly(ethylene oxide) backbone (PDMS_36_‐*b*‐PEO_23_) and a cationic switchable lipid (CSL).^[^ ^221^ ^]^ Acidification induced a conformational change of CSL which resulted in a membrane destabilization allowing for dynamic changes in hGUV morphology. Recently, pH‐responsive poly(ethylene oxide)‐*block*‐poly[2‐(diisopropylamino)ethyl methacrylate] (PEO_45_‐*b*‐PDPA_20_) was used in combination with commercial poly(ethylene oxide)‐*b*‐poly(1,2‐butadiene) (PEO_34_‐*b*‐PBD_46_) to produce homogeneous, purely polymer GUVs by microfluidics.^[^ ^222^ ^]^ Confocal microscopy revealed that these p‐GUVs were stable at physiological pH (pH 7.4) and responded to small changes in pH by disruption. Another environmental factor regulating biochemical reaction networks is temperature. The effect of temperature on the assembly of individual GUVs, has been recently studied albeit with lipidic GUVs.^[^ ^223^ ^]^ In one of the few examples of temperature responsive GUVs assembled from synthetic polymers, polybutadiene‐poly(ethylene oxide) GUVs were formed with a temperature‐responsive poly(*N*,*N*‐dimethylacrylamide)‐*b*‐poly(N‐isopropylacrylamide) (PDMA‐*b*‐PNIPAM) diblock copolymer below the lower critical solution temperature of NIPAM.^[^ ^224^ ^]^ When the temperature was increased, the NIPAM block formed undefined polymeric structures that changed vesicle membrane dynamics. However, this study did not specifically address any downstream process in response to the temperature trigger. In contrast, light‐induced downstream activity of encapsulated enzymes was reported in poly(butadiene)‐*block*‐poly(ethylene oxide) (PB_22_‐*b*‐PEO_12_) GUVs where controlled permeability to external molecular signals was achieved by integrating spiropyran‐based modulators into the membrane (**Figure** **11**).^[^ ^225^ ^]^ The light‐triggered permeability afforded the entry of substrate into the GUVs where its hydrolysis was then catalyzed by the resident enzyme. Moreover, upon entry of ATP, coacervate droplets were formed inside photoactivated PB_22_‐*b*‐PEO_12_ GUVs encapsulating poly‐l‐lysine, reminiscent of the adaptive formation biological condensates in natural cells. The limitation associated with direct membrane passage is that it is rather unselective and only very small molecules can diffuse across the membrane, which also is more prone to disruption.

Several strategies exist to overcome the characteristic low permeability of polymer membranes in order to have them more closely mimic the adaptive behavior of their biological counterparts. One strategy is to design membranes from “leaky” polymers or from polymers that change their properties in response to an environmental signal. A microfluidic approach was used to generate p‐GUVs from different molar ratios of triblock PEO_5_‐*b*‐PPO_67_‐*b*‐PEO_5_, PEO_4_‐*b*‐PPO_58_‐*b*‐PEO_4_, and PEO_2_‐*b*‐PPO_30_‐*b*‐PE_2_ that showed a differential permeability to ions and small dye molecules, depending on the thickness of the membrane which was decreased by blending in polymers with a shorter hydrophobic block.^[^ ^79^ ^]^ Rehydration of thin films from PMOXA_5_‐*b*‐PDMS_58_‐*b*‐PMOXA_5_ with 8 wt% PDMS_65_‐*b*‐heparin has resulted in pGUVs that were permeable to the small‐molecule redox reagent dithiothreitol (DTT) but not to the tripeptide glutathione which is an essential intracellular reductant.^[^ ^15^ ^]^ As described further up, externally added DTT was able to activate a cascade of reactions involving several subcompartments inside of the GUV. The limitation of this system is that signal transfer depends on the passive diffusion which is a rather slow and inefficient process.

Alternatively, transmission of external signals across the polymer membrane can take place via peptide and protein biopores which provide a gateway for larger molecules such as resorufin or glucose to diffuse into GUVs.^[^ ^20^, ^119^, ^226^ ^]^ Again, these communication pathways posed restrictions on the molecular size of signals and were regulated by passive diffusion. Larger communication channels were recently obtained by DNA origami pores inserted in p‐GUVs,^[^ ^122^, ^123^ ^]^ and l‐GUVs.^[^ ^41^ ^]^ For l‐GUVs, the membrane spanning DNA origami signaling units were mechanically coupled to an internal DNA cytoskeleton mimic and chemical signal transduction of an invader DNA induced the disassembly of the DNA cytoskeleton inside the GUV.^[^ ^41^ ^]^

Although nature makes use of active transport, e.g. proton pumps to ferry signaling molecules across membranes at a high flux,^[^ ^227^ ^]^ integrating these features in artificial membranes remains challenging. There are only few examples so far of functional reconstitution of active transporters (e.g., proton pumps) or other transmembrane proteins that undergo conformational changes to transmit information across the GUV membrane without transporting the signaling molecule itself, and in most cases integration was successful in lipid‐based artificial cells.^[^ ^9^, ^41^, ^228^, ^229^, ^230^, ^231^ ^]^

Two recent studies, both using integration of DNA structures in lipid‐based artificial cells, represent first steps toward a mechanical signaling pathway that does not involve the physical transfer of mass across the artificial cell membrane.^[^ ^41^, ^232^ ^]^ In one report, a DNA‐based transmembrane pore was engineered such as to bind and rearrange preformed DNA filament structures residing in the GUV cavity when induced to cluster in the membrane by addition of streptavidin to the external surrounding.^[^ ^41^ ^]^ Similarly, by lowering the external pH, DNA origami‐based artificial membrane receptors were induced to dimerize which led to an amplification of fluorescence inside the artificial cell.^[^ ^232^ ^]^

### 5.2 Mimicking Cell Shape Changes in p‐GUVs

Shape plays a fundamental role in many cellular functions including growth and division, apoptosis, and motility.^[^ ^233^ ^]^ As functional units of a tissue, shape changes at the cellular level are important determinants of development and are also associated with many pathological conditions such as cancer. Many communication pathways in eukaryotic cells involve shape changes, frequently based on cytoskeleton dynamics. Owing to the ability to control the composition of the membrane and that of the inside and outside environment, biomimetic systems lend themselves to study shape changes in a less complex environment than the cytoplasm. The majority of studies addressing cell shape changes in GUVs are carried out using lipidic GUVs and we refer the reader to a recent review for an overview on actin‐induced cell shape changes in l‐GUVs.^[^ ^234^ ^]^

While cell division is essential for life, division (also referred to as budding) has so far been studied primarily in lipid‐based vesicles. For example, budding of phospholipid vesicles has been demonstrated to occur under various conditions, such as mechanical shearing^[^ ^235^, ^236^ ^]^ and temperature changes.^[^ ^237^ ^]^ The dynamic membrane allowed for phase separation and division of lipidic GUVs.^[^ ^238^, ^239^ ^]^ In another study, giant phospholipid vesicles underwent growth and budding followed by daughter vesicle formation by exploiting phospholipid synthesis and the production of additional membrane surface via a copper(I)‐catalyzed azide‐alkyne cycloaddition (CuAAC) reaction.^[^ ^240^ ^]^

Because the robust nature of polymer membranes makes it more difficult to induce dynamic shape changes in p‐GUVs, not to mention division, reports on shape changes of p‐GUVs are less frequent.^[^ ^241^ ^]^ On the other hand, p‐GUVs were shown to be considerably more stable in response to mechanical stress than the more deformable l‐GUVs^[^ ^34^ ^]^ and corresponding membrane modifications provide polymeric membranes with more flexibility.^[^ ^245^ ^]^ The predominant methods for inducing shape changes in polymer‐based compartments involve either a significant transmembrane osmotic imbalance or changes in the physiochemical properties of the membrane constituents.^[^ ^148^ ^]^ Accordingly, nano‐sized spherical polymersomes were shown to undergo shape transformation into tubular ones under hypertonic conditions or chemical modification of the polymeric bilayer.^[^ ^246^ ^]^ Shape changes were also induced by reconstitution of a correctly oriented, minimal SNARE fusion machinery into the membrane of polymersomes and lipid‐polymer hybrid vesicles, ultimately resulting in the fusion of the vesicles.^[^ ^247^ ^]^

At the micron length scale, the deformation of poly(butadiene)‐*b*‐poly(ethylene oxide)(PB‐*b*‐PEO) GUVs with small tubular extensions resulting from an increase of osmotic pressure inside the GUVs culminated in the formation of two connected vesicles.^[^ ^31^ ^]^ Morphological changes were also observed in rather small p‐GUVs (≈1.4 µm) produced by a microfluidic approach from a temperature sensitive triblock poly(*N*‐vinylcaprolactam)_15_‐*b*‐poly(dimethylsiloxane)_65_‐*b*‐poly(*N*‐vinylcapro‐lactam)_15_ triblock copolymer (PVCL_15_‐*b*‐PDMS65‐*b*‐PVCL_15_).^[^ ^248^ ^]^ At 42 °C, where PNVCL exhibits lower critical solution temperature (LCST) behavior, the collapse of PVCL blocks led to the onset of membrane buckling which facilitated the segmentation of bent membrane into multiple small vesicles.

**Figure 12:**
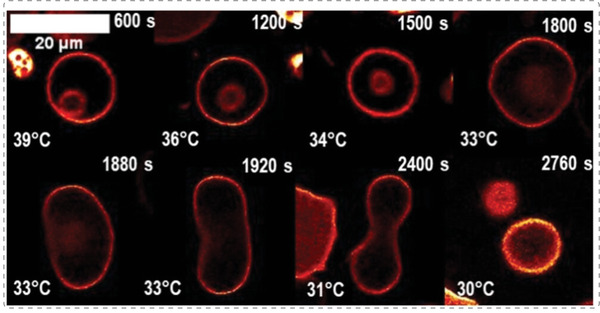
Self‐division in polymersome during cooling from 39 to 28 °C observed by confocal microscopy. PDMA_30_‐*b*‐PNIPAM_200_‐BODIPY:PDMA_30_‐*b*‐PNIPAM_200_ (1 : 100) (90 mg mL^−1^) and membrane PBD_51_‐*b*‐PEO_27_. Reproduced with permission under terms of the CC‐BY‐NC license.^[^ ^224^ ^]^ Copyright 2022, Wiley‐VCH.

Cross‐linked diblock copolymer PEO‐*b*‐P(NIPAAm‐r‐NAPMAm‐r‐NAPMAmRu(bpy)3‐r‐NAPMAmMA) exhibited temperature dependent swelling/deswelling cycles that were associated with unique shape changes.^[^ ^249^ ^]^ With the stepwise temperature decrease, the p‐GUV membrane experienced tangential stress caused by the expansion of the NIPAAm thermo‐responsive segment through hydration. To relax this sudden stress, the membrane temporarily buckled inward to increase its surface area. After the transient buckling deformation, the p‐GUV gradually relaxed to an unbuckled equilibrium state with a larger diameter. Similarly, in p‐GUVs formed from a temperature‐responsive diblock copolymer, poly(*N*,*N*‐dimethylacrylamide)‐block‐poly(*N*‐isopropylacrylamide) (PDMA‐*b*‐PNIPAM) and non‐responsive poly(butadiene)‐*b*‐poly(ethylene oxide) (PBD‐*b*‐PEO) via a double emulsification method at room temperature, that is, below the LCST of PNIPAM (32 °C in aqueous solution), the NIPAM block changed its colloidal properties within the membrane upon increasing the temperature.^[^ ^224^ ^]^ The resulting mechanical fluctuations in the polymer induced morphological changes of the p‐GUVs. Relaxation of PDMA‐*b*‐PNIPAM upon cooling, associated with an increase of the volume fraction, produced a range of morphology changes including occasional self‐divisions (**Figure** **12**).

Opposite to budding, mechanically activated fusion of nanometer‐sized vesicles made of poly(dimethylsiloxane)‐poly(ethylene oxide) graft copolymer (PDMS‐*g*‐PEO) with p‐GUVs under physiological salt conditions has been observed, which resulted in growth of the GUVs.^[^ ^250^ ^]^ Hybrid vesicles made of the same polymer and oppositely charged lipids also underwent rapid charge‐mediated fusion. Importantly, bo3 oxidase reconstituted into small (<1 µm) cationic hybrid vesicles was efficiently transferred to the membrane of anionic hybrid GUVs trapped in microfluidic device by charge‐mediated fusion.

In natural cells, a plethora of functions depend on a dynamic cytoskeletal framework which at the same time provides the cells' structure. The actin cytoskeleton in particular is a key driver of shape changes^[^ ^251^, ^252^ ^]^ but also plays an important role in signaling^[^ ^253^ ^]^ and other membrane‐related processes such as endo/exocytosis.^[^ ^254^ ^]^

Some features of cytoskeletal shape control could be minimally re‐constituted by encapsulating individual filamentous systems inside l‐GUVs.^[^ ^32^, ^255^, ^256^, ^257^, ^258^, ^259^ ^]^ When confined in l‐GUVs, actin, α‐actinin, and fascin (an actin bundling protein) formed complex structures, including rings and asters at GUV peripheries and centers whereby the prevalence of different structures depended on GUV size.^[^ ^260^ ^]^ A more complex setup using a lipidic GUV/proteoliposome system demonstrated successful coupling of photosynthesis and actin polymerization.^[^ ^261^ ^]^ To address the complex mechanism of cytoskeleton formation inside p‐GUVs, monomeric actin was encapsulated together with filamin, an actin crosslinking protein, in GUVs produced by film rehydration of PMOXA_5_‐*b*‐PDMS_58_‐*b*‐PMOXA_5_ and PDMS_65_‐*b*‐heparin copolymers (Figure 4).^[^ ^34^ ^]^ Polymerization of actin into crosslinked filaments and filament bundles was triggered either by adding salt together with corresponding ionophores to the GUV environment or if ionophores were not added from the outside, by a signaling cascade that prompted the release of ionophores into the p‐GUV cavity from subcompartments co‐encapsulated with monomeric actin and filamin. Ionophores then inserted into the p‐GUV membrane from the inside and enabled influx of ions from the environment that induced actin polymerization.

### 5.3 Communication between Artificial Cells

Bottom–up engineering enables the construction of artificial cells that are specifically designed for building controlled communication pathways while avoiding nature's complexity of cell–cell and cell–extracellular matrix interactions. The first step toward engineering molecular communication networks between protocells requires signals originating from a sender protocell to be transmitted to and processed by the receiver (Figure 10b). In the simple, binary scenario, the sender generates a diffusible chemical messenger in situ that is transmitted to a nearby receiver cell where it triggers a response by direct interaction with its cognate regulator. Signal propagation is usually unidirectional and involves passive diffusion from sender to receiver, afforded either by an intrinsically porous membrane^[^ ^19^ ^]^ or by communication channels (e.g., α‐hemolysin or OmpF) integrated into the membrane,^[^ ^119^, ^262^ ^]^ and thus is affected by the distance between the cells.^[^ ^263^ ^]^ Accordingly, the defined binary assembly of GUVs has been facilitated by linking two kinds of vesicles via complementary DNA (e.g., refs. [264, 265]). Optical tweezers have also been used to bring together artificial cells in a specific manner.^[^ ^266^ ^]^ GUVs composed of phospholipids have been used most commonly to study communication between two populations of artificial cells, mainly because their close‐to‐nature membrane can be more easily modified to facilitate diffusion of chemical signals. Because our focus lies on polymer‐based artificial cells we refer to other reviews covering communication between lipidic artificial cells (e.g., refs. [263, 267, 268]) for a more comprehensive overview and here present selected examples where polymeric entities like coacervates are involved in communication. Importantly, polymer‐based protocells might be advantageous to overcome one of the main drawbacks of l‐GUVs which is that they are generally very fragile and imbalanced osmotic conditions can easily cause their deformation or rupture,^[^ ^269^, ^270^ ^]^ impeding their application in advanced protocell development.

**Figure 13:**
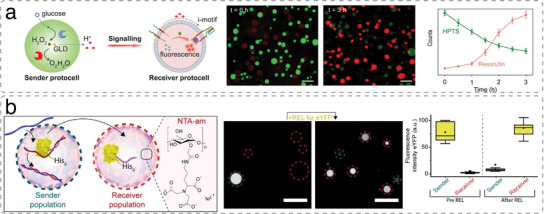
Cell–Cell communication between two artificial cells. a) Fluorescence‐activated signal communication behavior between two populations of synthetic protocells, based on low pH‐mediated dimerization of transmembrane DNA receptors. Left: Cartoon of coacervate microdroplets encapsulated with GOx/CAT enzyme that serve as sender protocells capable of H+ release in the presence of glucose. L‐GUVs decorated with pH‐responsive artificial receptors serve as receiver protocells capable of H+‐mediated signal transduction and fluorescence signal amplification inside the l‐GUVs through an intravesicular peroxidase‐like cascade reaction. Middle: Fluorescent confocal imaging of binary populations shows the signal communications between sender (green domain) and receiver (red domain) protocells, upon the addition of glucose for *t* = 0 and 180 min. Scale bar: 30 µm. Right: Mean fluorescence intensity plots of a mixed protocell population interacting for 180 min, showing the decrease of HPTS fluorescence (green) for sender protocells and the correlated increase of resorufin fluorescence (red) from Amplex Red oxidation in receiver protocells. Reproduced with permission.^[^ ^232^ ^]^ Copyright 2023, Wiley‐VCH. b) Left: Schematic showing protein shuttling between two different coacervate‐based artificial cells. The sender population was loaded with eYFP and UPT strand, the receiver population was equipped with NTA‐amylose (NTA‐am) and Ni^2+^. Upon addition of REL strands, His‐tagged eYFP was released from the sender population and incorporated by the receiver population. Middle: Confocal laser scanning microscopy images showing the release and subsequent uptake of eYFP (100 nm) from the sender population (blue) to the receiver population (red). After adding the REL strand, the solution was incubated for 60 min at room temperature. The experiment was performed in PBS containing 5 mm MgCl_2_ and 3.75 µm NiCl_2_, pH 7.4, *I* = 185 mm. Right: Box plots displaying the quantified fluorescence intensity of eYFP inside the sender and receiver artificial cell population before and after addition of the REL strand. Fluorescence intensity was determined inside the core of ≥15 coacervates. ▪ represents the mean, ♦ represents outliers. Scale bars represent 20 µm. Reproduced under the terms of the CC‐BY license.^[^ ^271^ ^]^ Copyright 2022, Wiley‐VCH.

In the DNA‐based artificial transmembrane signal transduction system described above,^[^ ^232^ ^]^ low external pH induced receptor dimerization was coupled to catalysis inside the lipidic GUV, resulting in the in situ formation of polydopamine in the absence of physical mass exchange. Signaling‐competent l‐GUVs were shown to establish signaling between poly(diallyl‐dimethylammonium chloride) PDD/DNA coacervate droplets sequestering GOx and catalase (sender) and the membrane‐bound protocells (receiver) (**Figure** **13**a). Addition of glucose to the combined protocells led to an acidification of the extracellular environment by the droplets which in turn induced receptor‐mediated polydopamine formation in the l‐GUVs. As transmembrane signal transduction has not been yet reported in p‐GUVs, such l‐GUVs set an example how the field can evolve, benefiting from mimicking natural processes to support advanced applications in polymeric systems.

In another study, protein shuttling between two different synthetic entities was addressed by exploiting complementary DNA elements for programmable control of protein transport across permeable membranes (Figure 13b).^[^ ^271^ ^]^ Here, a triblock copolymer PEG‐PCL*g*TMC‐PGlu (poly(ethylene glycol)‐poly‐(caprolactone‐gradient‐trimethylene carbonate)‐poly‐(glutamic acid)) was self‐assembled on amylose‐based coacervate droplets to form a stabilizing semi‐permeable membrane. The positive charge of this artificial cell platform allowed the sequestration of negatively charged proteins such as eYFP, and fluorescently labeled myoglobin and horseradish peroxidase. To enable uptake of these proteins, they were conjugated to a 12‐mer single‐stranded oligonucleotide to which a partially complementary 32‐mer termed UPT strand (uptake strand) was hybridized, providing the tagged proteins with sufficient negative charge to trigger their partitioning in the coacervates. Interestingly, the sequestration could be reversed by adding a releaser strand (REL) that was complementary over the entire length of the UPT. The DNA handle allowed to control the protein release by using different amounts of REL and modifying the sequence complementarity. By using a set of different orthogonal combinations of UPT‐REL, temporal control over the release of a specific protein was achieved. Protein shuttling between artificial coacervate‐based cells was demonstrated by triggering a sender population sequestering a His‐tagged 12nt‐ssDNA‐eYFP to release a His‐tagged protein that was captured by a receiver population where the coacervate matrix was equipped with NTA‐amylose (Figure 13b).

3D architecture and the ensuing cell–cell and cell–ECM interactions play a fundamental role in tissue/organ function. To more closely mimic tissue organization, bottom–up synthetic biology is increasingly directing its attention to spatially encoded systems. Such interactions have been reported by taking as model two types of individual l‐GUV suspensions, each exhibiting a different fluorescence, which could be magnetically gathered into colonies with different morphologies and spatial organizations via the modulation of SS mesh parameters (microwell morphologies and organizations) and external magnetic field.^[^ ^272^ ^]^ Interestingly, these l‐GUV colonies exhibited an unexpected stability toward osmotic imbalance compared to the individual, fragile l‐GUVs. The closely packed GUVs in the colony acted as a collective to resist the external osmotic shock. Hypertonic osmotic stress compressed the GUV colony further which increased the elasticity energy of the colony, resulting in a negative hydrostatic energy that was released by the entering of water into the colony. The negative hydrostatic energy (promoting water in and elasticity energy release) balanced the osmotic stress (promoting water out) to stabilize the GUVs colony under hypertonic conditions.

To mimic extracellular matrix interactions in prototissue formation, a framework of DNA‐nanotubes and fibers was recently attached via cholesterol anchors to the surface of lipidic protocells.^[^ ^11^ ^]^ Upon evaporation‐induced convection and weak electrostatics, l‐GUVs dispersed in an aqueous‐glucose droplet on a glass surface assembled prototissues, which could be further cross‐linked by adding more interacting DNA nanotubes or fibers to obtain prototissue with different morphology and connectivity. By using polymer compartments in a likewise manner, multifunctional advanced systems with increased robustness are to be expected.

### 5.4 Tackling Collective Behavior

**Figure 14:**
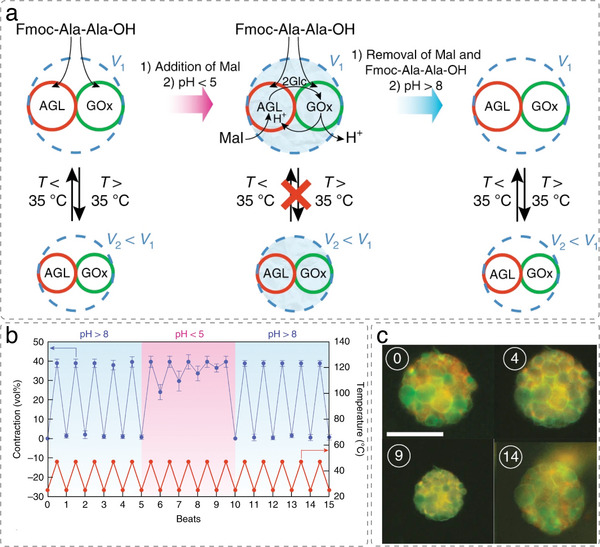
Enzyme‐mediated amplitude modulation within thermoresponsive prototissue spheroids. a) Scheme representing the coupling of contractile behavior to an AGL/GOx cascade reaction within a prototissue spheroid (blue dashed circle) consisting of bio‐orthogonally linked AGL‐containing RITC‐labelled azide‐functionalized proteinosomes (red circles) and GOx‐containing FITC‐labelled alkyne‐functionalized proteinosomes (green circles). The prototissue reversibly contracts/relaxes in the presence of peptide Fmoc‐Ala‐Ala‐OH at pH 8.5 (left). Addition of maltose (Mal) triggers the enzyme cascade and reduces the pH below 5, initiating peptide hydrogelation, which hinders re‐expansion (middle). Removal of Mal and Fmoc‐Ala‐Ala‐OH, and restoration of an alkaline pH disassemble the hydrogel and re‐establishes reversible contractile behavior (right). b) Graph showing amplitude modulation of contractile behavior of uncaged prototissue spheroids for the conditions described in a for repeated thermal cycling between 25 and 47 °C (blue plot). Corresponding cycles in temperature are shown in red. c) Time‐dependent fluorescence microscopy images acquired at 25 °C of a single prototissue spheroid subjected to the conditions described in a and b, showing switching off and on of reversible contractile behavior. Red fluorescence, AGL‐containing RITC‐labelled azide‐functionalized proteinosomes. Green fluorescence, GOx‐containing alkyne‐functionalized proteinosomes. The beat number is indicated at the top left of each image, and corresponds to the graph in (b). Scale bar, 50 µm. Reproduced with permission.^[^ ^58^ ^]^ Copyright 2018, Springer Nature.

A major challenge in developing tissue‐mimetic protocell communities with different functionalities is to direct communication while taking into account increased crosstalk and interference associated with different types of sender–receiver pairs (Figure 13). Hitherto, the majority of studies tackling collective behavior apply lipidic GUVs in combination with polymeric entities such as coacervates.^[^ ^232^, ^273^, ^274^, ^275^ ^]^ Protocell–protocell communication within a spatially confined ensemble of individual protocells that is able to sense chemical stimuli (glucose) in the environment was also reported for a community of bio‐orthogonally linked proteinosome‐based protocells capable of thermo‐responsive collective behaviors, that is, enzymatically modulated reversible contraction and rudimentary mechanochemical transduction.^[^ ^58^ ^]^ The researchers produced synthetic spheroids (i.e., self‐assembling cell aggregates), from two types of proteinosomes that were spatially confined in water/oil/water droplets and then linked via interfacial strain‐promoted alkyne‐azide cycloaddition reaction (I‐SPAAC). By this approach, caged and uncaged spheroids with an average size of 70 µm and 120 µm, respectively, could be prepared that comprised 20–90 interlinked proteinosomes. Owing to the thermo‐responsive poly(*N*‐isopropylacrylamide‐*co*‐methacrylic acid) (PNIPAM‐*co*‐MAA), both caged (by a non‐bio‐orthogonally linked outer BSA/PNIPAM) and uncaged spheroids reversibly undergo at least ten contraction/relaxation cycles, manifest by a change in volume, in response to externally applied temperature transitions above and below the lower critical solution temperature (LSCT). The contraction/relaxation of the simple prototissues could be coupled to an α‐glucosidase/glucose oxidase cascade reaction that took place between linked proteinosomes in the presence of maltose substrate (**Figure** **14**). Collective interactions and coordinated responses are brought about by bio‐orthogonal, semipermeable protein–polymer biocapsules spatially integrated within the synthetic tissue‐like spheroids.

In natural cell communities, the spatial distribution of senders and receiver is an important factor determining specificity of communication. In a recent study, the authors control which sender–receiver pairs communicate in a three‐membered community of proteinosomes through red and blue light illumination.^[^ ^276^ ^]^ The semipermeable proteinosomes, one sender and two receivers, differed by their surface modification with complementary protein adhesion molecules that are triggered to interact by light at different wavelengths. Light regulated proximity controlled the communication, however, the signaling range of the sender population was limited to nearest neighbor interactions. Clearly, the 3D architecture of synthetic cell communities will need to be extensively explored in order to create prototissues with life‐like functionality and adaptivity.

## 6 Conclusion and Outlook

Artificial cells constructed through bottom–up approaches hold great potential in advancing the understanding of life as well as in technological and biomedical applications, including compound production, detoxification from harmful substances, or protein replacement therapy. The building blocks span a large variety of micro‐ and nanoscale assemblies of different chemical nature (lipids, peptides, polymers) in combination with biomolecules and active compounds. In this review, we focused on approaches to develop synthetic cells whose compartments have polymers as main constituents. We took this perspective due to the broad spectrum of polymers and resulting assemblies with fine‐tuned or even new‐to‐nature properties and functionalities that are expected to support the development of advanced multifunctional systems. However, the complexity of natural cells poses challenges in developing fully autonomous materials that can function as equal counterparts to biological cells. Here the advantages of the bottom–up strategy come into effect; cellular phenomena and processes can be simplified and studied under user‐defined conditions without the bustling environment of natural cells. At the same time, by incorporating different nano‐ and microcompartments into artificial cells, a multicompartment architecture can be created that promotes communicative reaction networks. Although the collection of functional modules used as building blocks for artificial cells has been rapidly expanding over the past years, they represent mainly model systems with simple architectures and/or reactions. Both, artificial organelles and cells are meant to provide insight into basic bio‐processes and cell‐related features. They are essential as an early‐stage research to prepare the next generation of artificial cells with more intricate metabolic pathways, intracellular interactions or the ability to repeatedly react stimuli and adapt to the respective environment. Strategies favoring a controlled arrangement of various organelles in a specific spatial context within cell‐like compartments are not yet in place. In addition, with compartments‐in‐compartment and coacervates more attention has been devoted to the biomimicry of 3D cell architecture than to the properties of individual polymer compartments aiming at medical applications, as for example biocompatibility and biodegradability. In this respect, synthesis routes providing polymers and assemblies that are able to more closely recapitulate in vivo features should be enlarged and optimized. More systematic studies are necessary to explore the fate of such polymer‐based artificial cells and move from single‐cell assays to animal models and beyond. Yet, the construction of synthetic prototissues, which involves assembling these protocells into complex micro‐architectures like spheroidal clusters and sheet‐like aggregates that can exhibit collective behavior, is still at its infancy. All current synthetic tissue‐mimics exhibit very limited stability, only few basic activities (linear communication and macroscopic deformation) and lack reversible responsiveness to external signals. A key strategy for achieving close‐to‐nature multifunctionality would be integrating a larger selection of more adept organelles and biologically relevant reactions inside artificial cells which then hierarchically organize into spatially defined multicellular entities. Advanced protocell materials that can interface with living cells and influence their behavior will break the ground for novel biomedical applications such as organoid formation, vasodilation, drug synthesis, and release. A step in this direction has been achieved by photo‐activation of conidiation in the fungus *Trichoderma atroviride* with Gaussia luciferase (Gluc)‐expressing synthetic cells.^[^ ^1^ ^]^

Despite significant technical progress in replicating features of living systems in artificial cells, integrating these features in life tissues remains challenging. Moreover, mimicking processes key to life are still riddled with bottlenecks, for example replicating information and ensuring cellular content following cell division.

The ultimate vision is to create synthetic cells capable of identifying and controllably treating diseases without adverse or off‐target effects. This approach holds great promise for repairing disturbed cell functions or compensating erroneous organelle division associated with diseases. To achieve these goals, an interdisciplinary approach is necessary, integrating basic and applied research in polymer chemistry, synthetic and molecular biology, cell physiology, biotechnology, and pharmacology. Concerted efforts will hopefully accelerate the clinical realization of many potential applications, ushering in a new era where polymeric artificial cells combined with advances in biotechnology and molecular biology lead to groundbreaking applications.

## Conflict of Interest

The authors declare no conflict of interest.
